# Schiff bases of sulphonamides as a new class of antifungal agent against multidrug‐resistant *Candida auris*

**DOI:** 10.1002/mbo3.1218

**Published:** 2021-07-23

**Authors:** Asad Hamad, Yiyuan Chen, Mohsin A. Khan, Shirin Jamshidi, Naima Saeed, Melanie Clifford, Charlotte Hind, J. Mark Sutton, Khondaker Miraz Rahman

**Affiliations:** ^1^ Department of Pharmacy The Islamia University of Bahawalpur Bahawalpur Pakistan; ^2^ Institute of Pharmaceutical Science King's College London London UK; ^3^ Public Health England National Infections Service Salisbury UK

**Keywords:** antifungal resistance, *Candida auris*, candidemia, Schiff bases, sulphonamides

## Abstract

Invasive *Candida* infections in hospitalized and immunocompromised or critically ill patients have become an important cause of morbidity and mortality. There are increasing reports of multidrug resistance in several *Candida* species that cause Candidemia, including *C*. *glabrata* and *C. auris*, with limited numbers of antifungal agents available to treat patients with invasive Candida infections. Therefore, there is an urgent need to discover new antifungal agents that work against multidrug‐resistant *Candida* species, particularly *C*. *auris*, which has been identified as an emerging global pathogen. In this article, we report a new class of antifungal agents, the Schiff bases of sulphonamides, that show activity against all *Candida* species tested, with an MIC range of 4–32 µg/ml. Compound **2b** showed activity against *C*. *glabrata* and a panel of fluconazole‐resistant *C*. *auris* strains, with MICs of 4–16 µg/ml. The drug‐like nature of these Schiff bases offers opportunities to optimize these compounds with medicinal chemistry techniques to obtain more potent analogs that can be progressed toward pre‐clinical evaluation.

## Graphical abstract

We report a new class of antifungal agents, the Schiff bases of sulphonamides, that show activity against all *Candida* species tested including multidrug‐resistant *C*. *auris*. The drug‐like nature of these Schiff bases offers opportunities to optimize these compounds with medicinal chemistry techniques to obtain more potent analogs that can be progressed toward pre‐clinical evaluation.

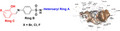

## 1 INTRODUCTION

The need for better antifungal therapy is urgent due to the high mortality rates that are associated with invasive fungal diseases, the limited number of effective antifungal classes, their associated toxicity, and the growing number of infections caused by multidrug‐resistant strains (Perlin et al., 2017). Coupled with a significant increase in the immunocompromised population, including those with HIV, cancer patients, and transplant recipients, this has led to a dramatic expansion of human invasive mycoses cases, particularly invasive candidiasis, which has become a global public health problem (Kullberg & Arendrup, 2015). A large number of resistant *Candida* species against first‐ and second‐line antifungal therapeutics, fluconazole, and echinocandins, for instance, are being reported in the clinic (Sanguinetti et al., 2015; Whaley et al., 2017). According to a report from the CDC, 7% of the strains were found to be resistant against fluconazole among all the *Candida* species isolated from the circulatory system. (Magill et al., 2014). Of those, 70% were identified as *Candida glabrata* and *Candida krusei*, which are intrinsically associated with innate resistance to various azole drugs (Pfaller & Diekema, 2007; Whaley et al., 2017). There are very few treatment options for multidrug‐resistant *Candida* infections as azoles and echinocandins remain the drug of choice despite their lack of efficacy as the remaining drugs have poor toxicity profiles and are often difficult to administer. (Arendrup & Patterson, 2017).

*Candida auris* is an emerging fungal pathogen that was firstly identified in Asia in 2009. According to data from the CDC, the case count was increased by 328% in 2018, and until August 2020, more than 1000 cases were reported (Bhattacharya et al., 2020). The major concerns for *C*. *auris* are its multidrug resistance, high mortality rate, difficulty in fast identification, and high risks of healthcare outbreaks (Nett, 2019). In 2020, several cases of hospital candidemia outbreaks related to COVID‐19 in Intensive Care Units and severe fungal co‐infections have been reported across the world including UK (White et al., 2020), India (Chowdhary et al., 2020), and China (Song et al., 2020). Treatment of candidiasis, including infections caused by *C*. *auris*, relies on very few classes of antifungal drugs. The vast majority of *C*. *auris* isolates sent to CDC possessed resistance to fluconazole and up to one third are resistant to amphotericin B (Borman et al., 2016). Therefore, there is an urgent need to identify new antifungal agents containing new chemical classes to treat drug‐resistant *Candida* infections.

Sulphonamides are a versatile class of drug‐like chemical scaffolds that have shown a wide range of therapeutic activities including antimicrobial (Seydel, 1968), antitumor (Bouissane et al., 2006), antiviral (Gawin et al., 2008), and anti‐inflammatory (Weber et al., 2004) effects. More recently sulphonamide derivatives like phosphodiesterase‐5 inhibitor sildenafil (Kim et al., 2001) are being used in the treatment of erectile dysfunction. In addition, a wide variety of sulphonamide derivatives have been evaluated as experimental agents against ulcerative colitis (Wilson et al., 2004), rheumatoid arthritis (Levin et al., 2002), obesity (Hu et al., 2001), anticancer agents (Ma et al., 2012), and in Alzheimer's disease (Roush et al., 1998). One of the widely employed techniques to explore the therapeutic utility of this chemical class is the conversion of sulphonamides into Schiff bases by condensing them with different aldehyde derivatives. The Schiff bases have been reported as anti‐infective agents including antimalarial (Rathelot et al., 1995), antimicrobial (Shi et al., 2007), antifungal (Guo et al., 2007), antiviral (Wang et al., 1990), anticonvulsant (Verma et al., 2004), and antiplasmodial (Adams et al., 2016) agents.

In this study, we explored the antifungal potential of three marketed sulfa drugs and their Schiff bases to identify new antifungal agents containing a sulphonamide chemical scaffold. While parent sulfa drugs were found to be inactive against all pathogenic *Candida* species tested, their Schiff bases showed promising antifungal activities against a panel of pathogenic *Candida* strains including an extended panel of multidrug‐resistant *C*. *auris*.

## 2 RESULTS AND DISCUSSION

**FIGURE 1:**
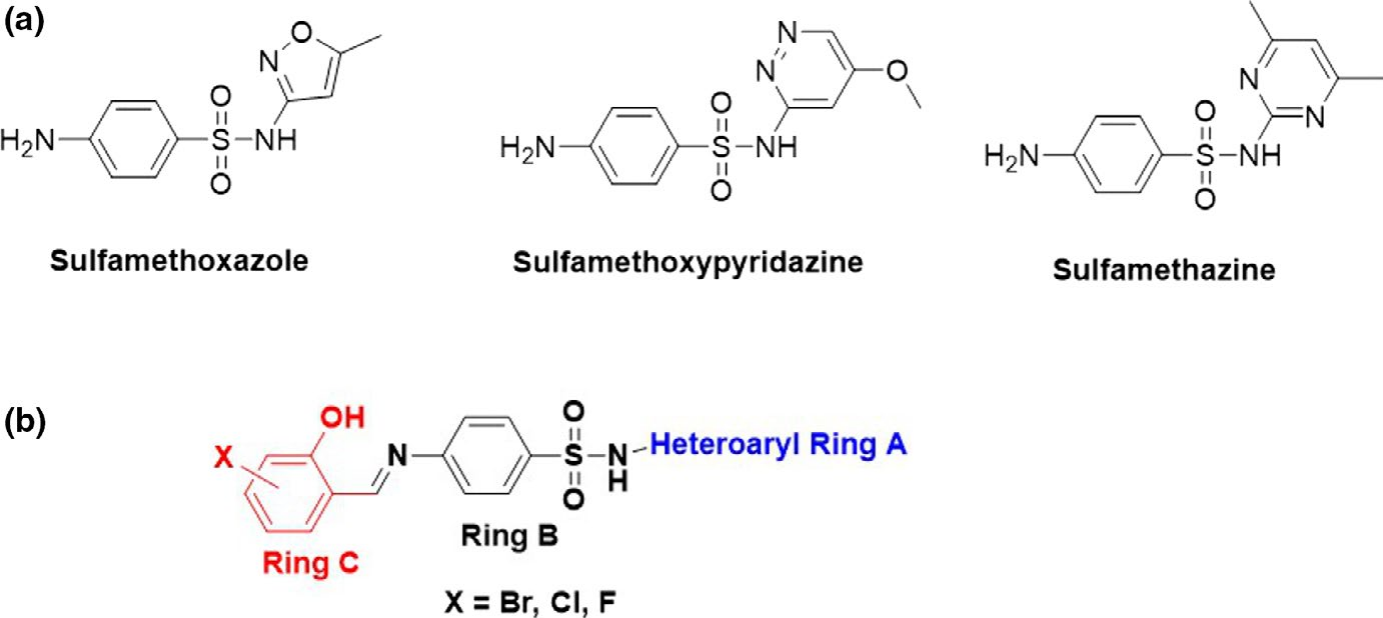
(a) Structures of sulfa drugs that were used to generate the Schiff bases; (b) General structure of the antifungal Schiff bases

**SCHEME 1:**
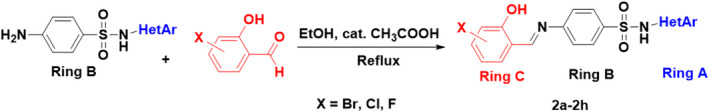
Reaction conditions for the synthesis of Schiff base derivatives (**2a‐2h**) of 4‐aminobenzenesulfonamide

The Schiff base derivatives of the sulfa drugs were synthesized by condensation of commercially available 4‐amino‐N‐(5‐methylisoxazol‐3‐yl)benzenesulfonamide (sulphamethoxazole), 4‐amino‐N‐(4,6‐dimethylpyrimidin‐2‐yl)benzenesulfonamide (sulfamethazine), and 4‐amino‐N‐(6‐methoxypyridazin‐3‐yl)benzenesulfonamide (Sulfamethoxypyridazine) with appropriate substituted aromatic aldehydes (Figure 1). A range of solvents with varying polarity was used to optimize the reaction conditions, and finally, ethanol with few drops of acetic acid was used as the optimum solvent mixture for the condensation reactions (Scheme 1). In every case, an equimolar quantity of sulphonamides and substituted aromatic aldehydes were used and the overall yield of the reactions ranged from 35 to 92%. The compounds were purified either by recrystallization or by liquid column chromatography prior to characterization, and all compounds were at least 95% pure before they were considered for microbiological evaluation. The structures of the synthesized Schiff bases were fully characterized with the help of ^1^H NMR, ^13^C NMR, and HRMS (ESI). Schiff bases of this type were previously reported by us (Hamad, Abbas Khan, et al., 2020; Hamad, Khan, et al., 2020), and the NMR data were compared with the literature, where applicable.

**FIGURE 2:**
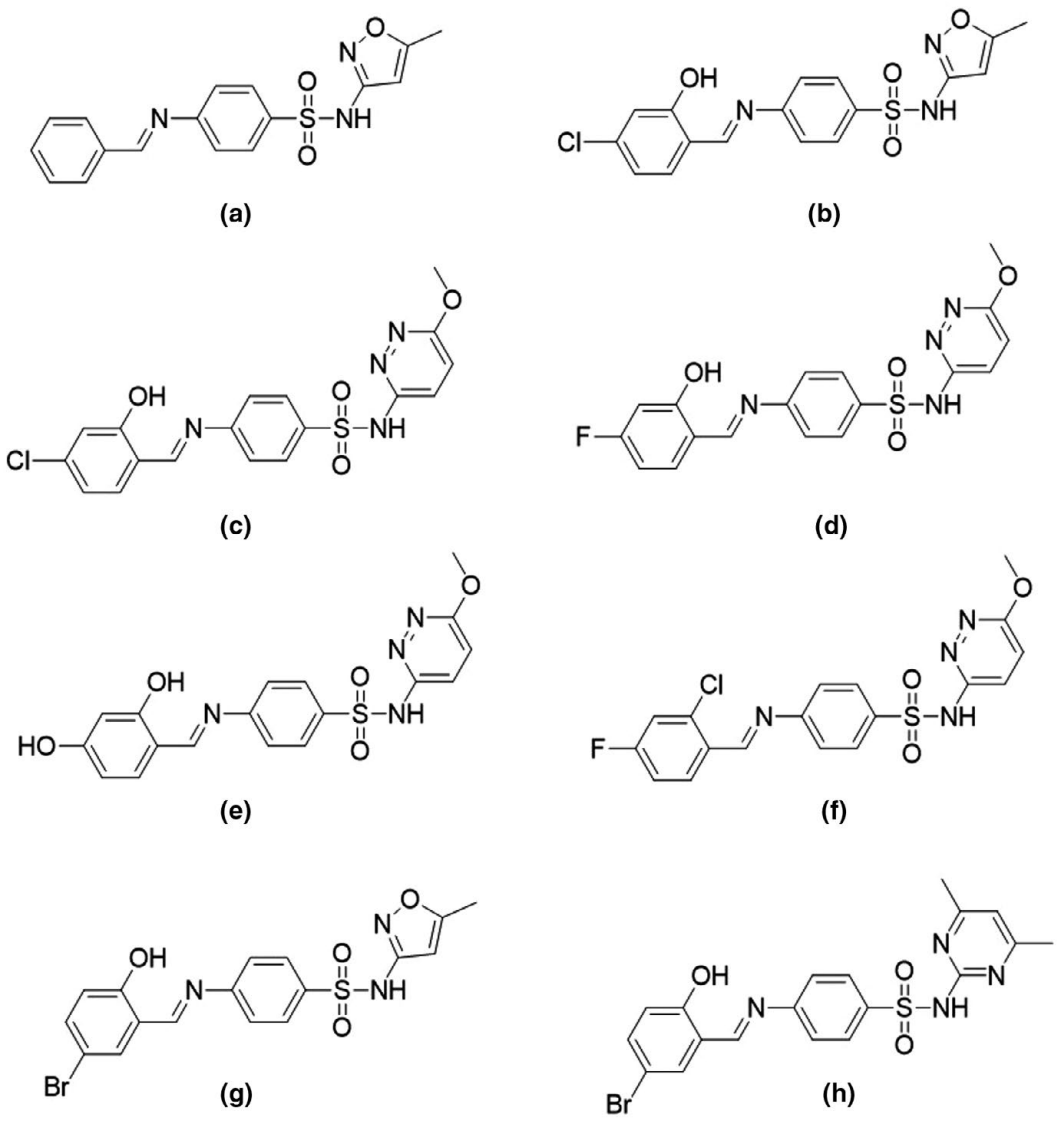
Structures of the Schiff bases evaluated for antifungal activities against the *Candida* panel

The synthesized compounds (Figure 2) were initially tested against a multi‐species panel of *Candida* strains to assess their antifungal activity. The panel consisted of *C. albicans* NCPF3281 and NCPF3179, *C*. *auris* TDG1912, *C. glabrata* NCPF8018, *C*. *krusei* NCPF3876, *C*. *tropicalis* NCPF8760, and *C. parapsilosis* NCPF3209 (Table 1). The Schiff bases that were found to be active against the *C*. *auris* strain TDG1912 were further tested against a larger panel of multidrug‐resistant *C*. *auris* strains (TDG2506, TDG2512, TDG1102, TDG2211, NCPF8984, NCPF8977, and NCPF8971) to confirm this activity (Table 2). All strains of the extended *C*. *auris* panel are clinical strains, except TDG2512 and TDG1912, which were environmental isolates but from hospital environments (Table S1). The panel represents three major clades (South Asian, East Asian, and South African) of *C*. *auris*.

**TABLE 1:** Antifungal activity of the Schiff bases against a multi‐species *Candida* panel

	Fluconazole	**2a**	**2b**	**2c**	**2d**	**2e**	**2f**	**2g**	**2h**
*C. auris* TDG1912	>128	>128	16	32	64–128	>128	>128	32	32
*C. albicans* NCPF3281	0.12 – 0.25	>128	16	32	32–64	>128	>128	32	32–64
*C. albicans* NCPF 3179	0.5	>128	16–32	32–64	64	>128	>128	64	64–128
*C. glabrata* NCPF8018	2	>128	4–8	16	16–32	>128	>128	8–16	16
*C. krusei* NCPF3876	16–32	>128	32–64	128	>128	>128	>128	64	64–128
*C. tropicalis* NCPF8760	8	>128	32	64	64–128	>128	>128	64	64
*C. parapsilosis* NCPF3209	0.12–0.25	>128	32–64	64–>128	64–>128	>128	>128	64	64–128

Note

MIC in µg/ml.

**TABLE 2:** Antifungal activity of selected Schiff bases against the extended *C*. *auris* panel

50% MIC	Fluconazole	**2b**	**2c**	**2d**	**2g**	**2h**
*C. auris* TDG2512	16	16	32–64	128	64	16
*C. auris* TDG1102	128	16–32	32	64–128	64	16–32
*C. auris* TDG2211	128	16–32	32–64	128	64–128	16–32
*C. auris* TDG2506	>128	16	32	32–64	32–64	16
*C. auris* NCPF8984	>128	16	32	64–128	32–64	16
*C. auris* NCPF8971	32	8–16	32	64	64	8–16
*C. auris* NCPF8977	32	16	32–64	64–128	64	16

Note

MIC in µg/ml.

All parent sulfa drugs (Figure 1a) were found to be completely inactive against the *Candida* strains with MIC values greater than 128 µg/ml. A Schiff base of sulfamethoxazole, **2a**, generated using benzaldehyde as the unsubstituted aromatic aldehyde was also found to be inactive against all strains tested. Intriguingly, the introduction of hydroxy and chlorine substitution on the aromatic aldehyde resulted in a Schiff base **2b** with antifungal activities against the *Candida* strains including the *C*. *auris* strain TDG1912 which is resistant to fluconazole (Table 1). The Schiff base **2b** had an MIC of 16 µg/ml against TDG1912, comparable to other *Candida* strains, except the *C. glabrata* strain NCPF8018 against which it had an MIC of 4–8 µg/ml, comparable to the activity of fluconazole. The Schiff base analog of sulfamethoxypyridazine, **2c**, containing the same aromatic aldehyde was synthesized to assess the importance of ring C (i.e., hydroxy and chloro‐substituted phenyl ring, Figure 1b) in conferring antifungal activity to the sulfa drugs. The Schiff base **2c** was also found to be active against the *Candida* strains with MICs ranging from 16 to 128 µg/ml. However, it was less active against all strains tested compared to **2b**, but more active than fluconazole against the *C*. *auris* strain TDG1912. This suggests the heteroaryl ring A also plays a role in antifungal activity in addition to ring C of these Schiff bases (Figure 1b).

To assess the effect of the electronegative halogen atom on the antifungal activity of the Schiff bases, compound **2d** was synthesized in which the chlorine was substituted with more electronegative fluorine. Both compounds **2c** and **2d** were Schiff bases of sulfamethoxypyridazine with the fluorine substitution in ring C in place of chlorine which allowed direct comparison of the activity of these two compounds. The Schiff base **2d** was slightly less active compared to **2c** against all *Candida* strains (MIC range 16 to >128 µg/ml), and it was found to be inactive against the *C*. *krusei* NCPF3876 strain. This suggests that the introduction of the more electronegative fluorine in ring C has a negative impact on antifungal activity for this chemical scaffold.

To assess the importance of halogen substitution in ring C of the Schiff bases, compound **2e** was synthesized in which the halogen atom in position‐4 was replaced with another hydroxy substituent. Interestingly, the compound was found to be inactive against all *Candida* strains tested (Table 1) suggesting the presence of the halogen atom in the ring is essential for antifungal activity. The Schiff base **2f** was synthesized to determine the effect of multiple halogen substitutions on activity. In this compound, the hydroxy group in position‐2 was replaced with a chlorine substituent allowing a direct comparison between **2d** and **2f**. Surprisingly, compound **2f** was also found to be completely inactive against all *Candida* strains, indicating the importance of the hydroxy group at position‐2 of the C‐ring.

Next, we evaluated the effect of the position of the halogen substitution on the activity of the Schiff bases. In compounds **2b**, **2c,** and **2d,** the 2‐position was substituted with a hydroxy group and the 4‐position was substituted with a halogen atom. Two Schiff bases **2g** and **2h** were synthesized with halogen substitutions on position‐5 of the ring while the hydroxy group was kept at position‐2. Both compounds **2g** and **2h** were active against all *Candida* strains with activity comparable to **2b** and **2c**. This suggests the position of the halogen substituent on the ring has a minor influence on the antifungal activity of the compounds.

After observing promising activity against the multi‐species *Candida* panel, we decided to focus more specifically on the ability of these Schiff bases to kill multidrug‐resistant *C*. *auris* strains. The Schiff bases **2b**‐**2e** were tested against an extended panel of fluconazole‐resistant *C*. *auris* strains. All Schiff bases were found to be active against the extended *C*. *auris* panel, with MICs ranging from 8 to 128 µg/ml (Table 2). The activity pattern of the compounds somewhat mirrored their activities against the multi‐species *Candida* panel with **2b** emerging as the most active compound with MICs in the range of 8–32 µg/ml. **2d** was least active with an MIC range of 32–128 µg/ml. Interestingly, the Schiff base **2h** with the halogen (bromine) substitution at the C5‐position was found to be more active compared to **2g**, and its activity against the *C*. *auris* panel was comparable to that observed for compound **2b**. Overall, the activity of these Schiff bases against the *C*. *auris* panel is encouraging and provides a new chemical scaffold to develop more potent antifungal agents against a pathogen of global concern. This is the first report of the sulphonamide class of antifungal agents with activity against *C*. *auris*.

The selectivity and therapeutic utility of these Schiff bases were further assessed using an MTT cytotoxicity assay against a non‐tumor lung fibroblast cell line WI38. The Schiff bases were tested at 40 and 80 µg/ml concentrations and were found to be non‐toxic (ESI) against this cell line at both concentrations tested (viability >90% at experimental conditions) suggesting a good selectivity for fungal pathogens.

**FIGURE 3:**
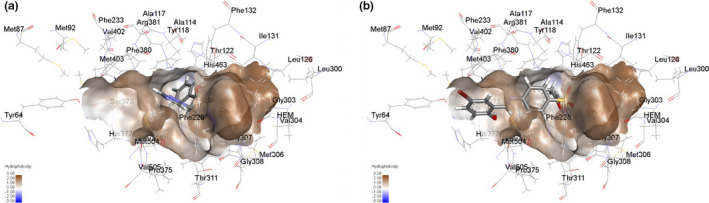
The Schiff **2b** occupies the azole binding pocket of wild‐type Erg11. (a) 3D molecular model showing fluconazole at the azole binding pocket and (b) 3D molecular model showing **2b** at the azole binding pocket

The mechanism of antifungal activities of these Schiff bases is not known. A molecular modeling study with the wild‐type and mutant Erg11 (F126L, F132Y, and K143R), the target enzyme for azole antifungals, showed the Schiff bases were capable of binding near the azole binding pocket of both the wild‐type and mutant Erg11 from *C*. *auris*. The molecular modeling study was conducted for compounds **2b**, **2c**, and **2h**, and all three ligands were able to bind to the azole binding pocket (Figure 3 and Figures S10‐S14) while the parent sulfa drugs did not interact with the Erg11. This suggests inhibition of Erg11 as a potential mechanism of action of these Schiff bases. However, more studies are required to determine the molecular mechanism of action of these Schiff bases against *C. auris*, which might initiate future drug discovery efforts using the Schiff bases of sulphonamides as a new antifungal chemical scaffold.

## 3 CONCLUSION

A new sulphonamide‐based chemical scaffold has been identified with broad‐spectrum antifungal activity against major *Candida* species, including multidrug‐resistant *C*. *auris* strains. The Schiff bases are non‐toxic against healthy human cell lines at the concentrations tested, which offers excellent opportunities to develop more potent analogs of this chemical class as antifungal agents. It was possible to establish a limited structure‐activity relationship that shows the importance of both halogen and hydroxy substituents on antifungal activity. Molecular modeling suggests Erg11 inhibition as a potential mechanism of action, but further work is necessary to determine the target and mechanism of this chemical class.

## 4 MATERIALS AND METHODS

### 4.1 General Experimental

All the solvents and reagents were commercially available from Sigma‐Aldrich, Fluorochem, Alfa Aesar, and Fisher Scientific, and were used directly without further purification. Melting points were measured with the Gallenkamp melting point apparatus. ^1^H and ^13^C nuclear magnetic resonance (NMR) spectra were obtained by a 400 MHz Bruker Spectrospin which was fitted with a Bruker SampleXpress autosampler system, and Topspin 7.1 was used for NMR spectra analysis. Chemical shifts of all compounds were calibrated with tetramethylsilane (TMS at δ = 0), with splittings outlined as singlet (s), doublet (d), and triplet (t). Fourier‐transform infrared (FTIR) spectroscopy was performed on Bruker TENSOR 27 FTIR spectrophotometer with the sample prepared using the KBr pellet press method. High‐resolution mass spectroscopy (HRMS) was performed using Agilent HP6890 GC with HP 7683 Injector interfaced directly to Agilent HP 5973 MSD (EI) instrument.

### 4.2 Chemistry

#### 4.2.1 General procedure for the synthesis of Schiff bases of 4‐aminobenzenesulfonamides

Sulphonamide, aromatic aldehyde, and glacial acetic acid were added to the absolute ethanol in a round‐bottomed flask, and the mixture was heated under reflux for 3 h, before cooling down to room temperature upon completion. The crude product was obtained by filtration, after which recrystallization was carried out for further purification. The product was dried overnight in the VacuumTherm (Thermo Scientific) vacuum oven prior to characterization. All final products were determined using mass spectroscopy and NMR.

##### 4‐(benzylideneamino)‐N‐(5‐methylisoxazol‐3‐yl)benzenesulphonamide (**2a**)

Physical appearance: Reddish Orange crystals; Reaction yield (80%); m. p. 197–199 ℃; Mol. Wt. 341.38; ^1^H NMR (DMSO−*d_6_*), δ ppm; 8.62 (s, 1H), 7.88 (d, 2H, *J* = 8.23 Hz), 7.76 (d, 2H, *J* = 7.78 Hz), 7.59 (t, 1H, *J* = 8.07 Hz), 7.56 (dd, 2H, *J* = 8.08 Hz, *J* = 2.02 Hz), 7.43 (d, 2H, *J* = 8.13 Hz), 6.05 (s, 1H), 3.23 (s, 1H, NH), 2.27 (s, 3H); ^13^C NMR (DMSO−*d_6_*), δ ppm; 139.23, 129.10, 122.34, 153.31, 149.34, 95.96, 169.77, 12.53, 159.47, 137.21, 128.98, 127.43, 132.22; HRMS (ESI‐MS, *m*/*z*): calculated for [C_17_H_15_N_3_O_3_S + H]^+^: 342.0900 found [M+H]^+^342.0907.

##### 4‐(4‐chloro‐2‐hydroxybenzylideneamino)‐N‐(5‐methylisoxazol‐3‐yl)benzenesulphonamide (**2b**)

Physical appearance: Reddish orange crystals; Reaction yield (78%); m. p. 189–191℃; Mol. Wt. 391.83; ^1^H NMR (DMSO−*d_6_*), δ ppm; 12.76 (s, 1H), 10.23 (s, 1H), 8.96 (s, 1H), 7.91 (d, 2H, *J* = 6.76 Hz), 7.58 (d, 1H, *J* = 8.12 Hz), 7.46 (d, 2H, *J* = 8.11 Hz), 7.08 (d, 1H, *J* = 8.03 Hz), 7.05 (s, 1H), 6.10 (s, 1H), 2.29 (s, 3H); ^13^C NMR (DMSO−*d_6_*), δ ppm; 138.69, 128.76, 122.72, 157.95, 153.00, 95.76, 170.33, 12.51, 161.43, 118.99, 134.04, 121.99, 140.81, 117.07, 164.67; HRMS (ESI, *m*/*z*): calculated for [C_17_H_14_ ClN_3_O_4_S + H]^+^: 392.0456 found [M+H]^+^392.0466.

##### 4‐{(*E*)‐[(4‐chlοro‐2‐hydroxyphenyl)methylidene]amino}‐*N*‐(6‐methοxypyridazin‐3‐yl)benzene‐1‐sulfοnamide (**2c**)

Physical appearance: Reddish orange crystals; Reaction yield (76%); m. p. 224–226℃; Mol. Wt. 418.85; ^1^H‐NMR (DMSO−*d_6_*), δ ppm; 8.97 (s, 1H), 7.90 (d, 2H, *J* = 6.69 Hz), 7.51 (d, 2H, *J* = 8.28 Hz), 7.46 (d, 1H, *J* = 6.98 Hz), 7.39 (d, 1H, *J* = 7.92 Hz), 7.05 (s, 1H), 6.57 (d, 1H, *J* = 8.04 Hz), 6.55 (d, 1H, *J* = 8.34 Hz), 5.92 (s, 1H), 3.87 (s, 1H), 3.85 (s, 3H); ^13^C NMR (DMSO−*d_6_*), δ ppm; 138.52, 128.03, 122.38, 156.34, 150.23, 120.14, 120.29, 157.40, 55.02, 161.32, 118.96, 161.32, 117.05, 140.11, 121.32, 134.11; HRMS (ESI, *m*/*z*): calculated for [C_18_H_15_ClN_4_O_4_S + H]^+^: 419.0567 found [M+H]^+^419.0575.

##### 4‐{(*E*)‐[(4‐fluοro‐2‐hydrοxyphenyl)methylidene]aminο}‐*N*‐(6‐methοxypyridazin‐3‐yl)benzene‐1‐sulfοnamide (**2d**)

Physical appearance: Reddish orange crystals; Reaction yield (79%); m. p. 209–211℃; Mol. Wt. 402.4, ^1^H‐NMR (DMSO−*d_6_*), δ ppm;, 8.97 (s, 1H), 7.92 (d, 2H, *J* = 6.63 Hz), 7.51 (d, 2H, *J* = 8.26 Hz), 7.42 (d, 1H, *J* = 6.97 Hz), 6.86 (d, 1H, *J* = 7.99 Hz), 6.84 (d, 1H, *J* = 8.09 Hz), 6.57 (d, 1H, *J* = 8.06 Hz), 6.55 (s, 1H), 5.95 (s, 1H), 3.87 (s, 1H), 3.84 (s, 3H); ^13^C NMR (DMSO−*d_6_*), δ ppm; 139.11, 128.04, 122.35, 155.82, 150.11, 120.51, 121.42, 158.34, 54.81, 162.97, 116.97, 162.97, 104.16, 167.07, 107.98, 135.27; HRMS (ESI, *m*/*z*): calculated for [C_18_H_15_ FN_4_O_4_S + H]^+^: 403.0863 found [M+H]^+^403.0871.

##### 4‐{(*E*)‐[(2,4‐dihydrοxyphenyl)methylidene]aminο}‐*N*‐(6‐methοxypyridazin‐3‐yl)benzene‐1‐sulfοnamide (**2e**)

Physical appearance: Reddish orange crystals; Reaction yield (78%); m. p. 201–203℃; Mol. Wt. 400.41; ^1^H‐NMR (DMSO−*d_6_*), δ ppm; 10.90 (s,1H), 10.40 (s, 1H), 8.82 (s, 1H), 7.86 (d, 2H, *J* = 6.83 Hz), 7.55 (d, 1H, *J* = 8.36 Hz), 7.52 (s, 1H), 7.39 (d, 2H, *J* = 7.87 Hz), 6.57 (d, 1H, *J* = 7.13 Hz), 6.55 (d, 1H, *J* = 7.23 Hz), 6.33 (d, 1H, *J* = 8.11 Hz), 3.87 (s, 1H), 3.84 (s, 3H); ^13^C NMR (DMSO−*d_6_*), δ ppm; 135.22, 128.04, 122.07, 156.33, 150.12, 120.51, 121.92, 163.55, 54.81, 163.62, 112.98, 163.70, 102.83, 164.66, 108.73, 133.20; HRMS (ESI, *m*/*z*): calculated for [C_18_H_16_N_4_O_5_S + H]^+^: 401.0905 found[M+H]^+^401.0914.

##### 4‐{(*E*)‐[(2‐chloro‐4‐fluοrophenyl)methylidene]amino}‐*N*‐(6‐methοxypyridazin‐3‐yl)benzene‐1‐sulphonamide (**2f**)

Physical appearance: Reddish orange crystals; Reaction yield (87%); m. p. 223–225℃; Mol. Wt. 420.85; ^1^H‐NMR (DMSO−*d_6_*), δ ppm; 8.80 (s, 1H), 7.88 (d, 2H, *J* = 6.95 Hz), 7.65 (d, 1H, *J* = 8.21 Hz), 7.38 (d, 2H, *J* = 8.17 Hz), 7.24 (d, 1H, *J* = 7.98 Hz), 6.85 (s, 1H), 6.57 (d, 1H, *J* = 7.29 Hz), 6.55 (d, 1H, *J* = 8.18 Hz), 3.87 (s, 1H), 3.84 (s, 3H); ^13^C NMR (DMSO−*d_6_*), δ ppm; 136.36, 128.00, 121.92, 154.33, 151.23, 120.23, 121.62, 159.91, 54.80, 157.85, 129.62, 136.91, 117.78, 165.52, 112.97, 133.36; HRMS (ESI, *m*/*z*): calculated for [C_18_H_14_ FClN_4_O_3_S + H]^+^: 421.0520 found [M+H]^+^421.0532.

##### 4‐(5‐bromo‐2‐hydroxybenzylideneamino)‐N‐(5‐methylisoxazol‐3‐yl)benzenesulfonamide (**2g**)

Physical appearance: Reddish orange red crystals; Reaction yield (81%); m. p. 199–201℃; Mol. Wt. 436.28; ^1^H NMR (DMSO−*d_6_*), δ ppm; 12.33 (s, 1H), 11.48 (s,1H), 8.91 (s, 1H), 7.94 (d, 2H, *J* = 6.99 Hz), 7.90 (d, 1H, *J* = 8.32 Hz), 7.56 (d, 2H, *J* = 8.41 Hz), 7.53 (d, 1H, *J* = 8.21 Hz), 6.99 (d, 1H, *J* = 8.17 Hz), 6.17 (s, 1H), 2.29 (s, 3H); ^13^C NMR (DMSO−*d_6_*), δ ppm; 138.95, 128.77, 122.71, 157.97, 153.21, 95.76, 170.33, 12.51, 160.32, 120.39, 134.04, 110.68, 136.69, 119.68, 163.98; HRMS (ESI, *m*/*z*): calculated for [C_17_H_14_ BrN_3_O_4_S + H]^+^: 435.9954 found [M+H]^+^435.9961.

##### 4‐(5‐bromo‐2‐hydroxybenzylideneamino)‐N‐(4,6‐dimethylpyrimidin‐2‐yl)benzenesulphonamide (**2h**)

Physical appearance: Reddish orange crystals; Reaction yield (83%); m. p. 211–213℃; Mol. Wt. 461.33; ^1^H NMR (DMSO−*d_6_*), δ ppm; 10.10 (s, 1H), 8.70 (s, 1H), 7.81 (d, 2H, *J* = 8. 41 Hz), 7.64 (d, 1H, *J* = 7.65 Hz), 7.44 (s, 1H), 7.42 (d, 2H, *J* = 8.02 Hz), 7.25 (d, 1H, *J* = 8.23 Hz), 6.74 (s, 1H), 3.91 (s, 1H), 2.25 (s, 6H); ^13^C NMR (DMSO−*d_6_*), δ ppm; 139.07, 129.90, 122.61, 156.57, 167.76, 166.81, 112.30, 23.52, 162.21, 121.34, 163.51, 121.62, 114.23, 125.50, 130.77 (C‐19); HRMS (ESI, *m*/*z*): calculated for [C_19_H_17_ BrN_4_O_3_S + H]^+^: 461.0269 found [M+H]^+^461.0278.

### 4.3 Determination of minimum inhibitory concentration

The MIC was determined according to modified EUCAST guidelines for azoles, echinocandins, and flucytosine in which we culture the test organisms overnights in liquid rather than on agar for MIC testing and adjust to the correct cell concentration using absorbance. Briefly, strains were grown overnight in RPMI‐1640‐MOPS containing 2% glucose and back‐diluted to a concentration of 0.5−5 × 10^5^ mCFU/ml. The back‐diluted cultures were added to a doubling dilution series of compounds (concentrations ranging from 128 µg/ml to 0.125 µg/ml) in non‐binding polystyrene 96‐well plates and incubated at 37℃ for 24 h. Wells were mixed by gentle pipetting, and then, MICs were defined as the lowest concentration of compound resulting in ≥50% growth inhibition compared to untreated drug‐free solvent controls.

### 4.4 Cell culture and MTT assay

The non‐tumor WI38 cell line was obtained from the American Type Culture Collection. The cells were maintained in a humidified atmosphere in an incubator at 37℃ containing 5% CO_2_. The cell line was maintained in Dulbecco's Modified Eagles Media (DMEM; Invitrogen) which was supplemented with fetal bovine serum (10% v/v; Invitrogen). The cells were plated in a 96‐well plate for the MTT viability assay, and the cells were incubated with the Schiff bases **2a‐2h** for 24 h. The MTT reagent was added to each well after the removal of media using an aspirator, and the formazan crystals were dissolved in DMSO. The absorbance values were read using an Envision plate reader (PerkinElmer). The values were normalized with the blank, and the normalized values were used for the determination of the % of viability. Each experiment was performed 6 times, and the result is reported as average values.

### 4.5 Molecular modeling

The 3D structures of wild type and F126L, F132Y, and K143R mutant ERG11 were obtained from homology modeling using the retrieved amino acid sequence from gene sequence with the code of A0A2H4QC40 (Template PDB id code: 5v5z, Seq. identity: 71.46%). Three forms of the mutations were generated using the PyMol program with an appropriate rotamer of mutated amino acid, which does not lead to any steric clash with the neighboring residues. All the structures were minimized and equilibrated using the AMBER program. Then molecular docking was performed by GOLD software by applying ChemScore as the scoring function. The cavity definition for the target protein was performed based on the known binding site of the corresponding crystal structures applied in this study.

## CONFLICT OF INTEREST

None declared.

## AUTHOR CONTRIBUTIONS

**Asad Hamad:** Formal analysis (supporting); Investigation (lead); Writing‐review & editing (supporting). **Yiyuan Chen:** Formal analysis (supporting); Investigation (supporting); Methodology (supporting); Project administration (supporting); Writing‐original draft (supporting). **Mohsin A. Khan:** Conceptualization (supporting); Funding acquisition (supporting); Methodology (supporting); Project administration (supporting); Supervision (supporting). **Shirin Jamshidi:** Investigation (supporting); Methodology (supporting); Writing‐review & editing (supporting). **Naima Saeed:** Formal analysis (supporting); Investigation (supporting); Writing‐review & editing (supporting). **Melanie Clifford:** Investigation (supporting). **Charlotte Hind:** Formal analysis (equal); Investigation (equal); Writing‐review & editing (supporting). **J. Mark Sutton:** Conceptualization (supporting); Funding acquisition (equal); Project administration (equal); Supervision (equal); Writing‐review & editing (supporting). **Khondaker Miraz Rahman:** Conceptualization (lead); Funding acquisition (equal); Project administration (lead); Supervision (lead); Writing‐original draft (lead); Writing‐review & editing (lead).

## ETHICS STATEMENT

None required.

## Supporting information

Supplementary Material

## Data Availability

All data generated or analyzed during this study are included in this published article and its supplementary material.
