# Supplementary material for: Schiff bases of sulphonamides as a new class of antifungal agent against multidrug‐resistant Candida auris
Source: Microbiologyopen. 2021 Jul 23;10(4):e1218. doi: 10.1002/mbo3.1218 (PMC8301596; doi:10.1002/mbo3.1218)
Supplement: Supplementary file 1 — Supplementary Material [file MBO3-10-e1218-s001.docx]

**Supporting Information**

# **Schiff bases of sulphonamides as a new class of antifungal agent against multidrug-resistant *Candida auris***

Asad Hamad^1,2^, Yiyuan Chen^2^, Mohsin A. Khan^1^, Shirin Jamshidi^2^, Naima Saeed^1^, Melanie Clifford^3^, Charlotte Hind^3^, J. Mark Sutton^3^* and Khondaker Miraz Rahman^2^*

^1^Department of Pharmacy, The Islamia University of Bahawalpur, Bahawalpur, Pakistan.

^2^Institute of Pharmaceutical Science, King’s College London, London, SE1 9NH, UK.

^3^Public Health England, National Infections Service, Porton Down, Salisbury, Wiltshire, SP4 0JG, UK.

**Table S1:** Source and details of clades of *Candida auris* strains used in the current study.

| Strain name | Clade | Source |
| --- | --- | --- |
| NCPF8984 | E. Asian | Clinical, unknown |
| NCPF8971 | S. Asian | Clinical, human wound swab |
| NCPF8977 | S. African | Clinical, human fluid |
| TDG1102 | S. African | Clinical, unknown |
| TDG2506 | E. Asian | Clinical, unknown |
| TDG2211 | S. Asian | Clinical, unknown |
| TDG2512 | S. Asian | Environmental |
| TDG1912 | S. African | Environmental |

**NMR and MS Spectra of Synthesized Compounds**

**4-(benzylideneamino)-N-(5-methylisoxazol-3-yl)benzenesulfonamide (2a)**

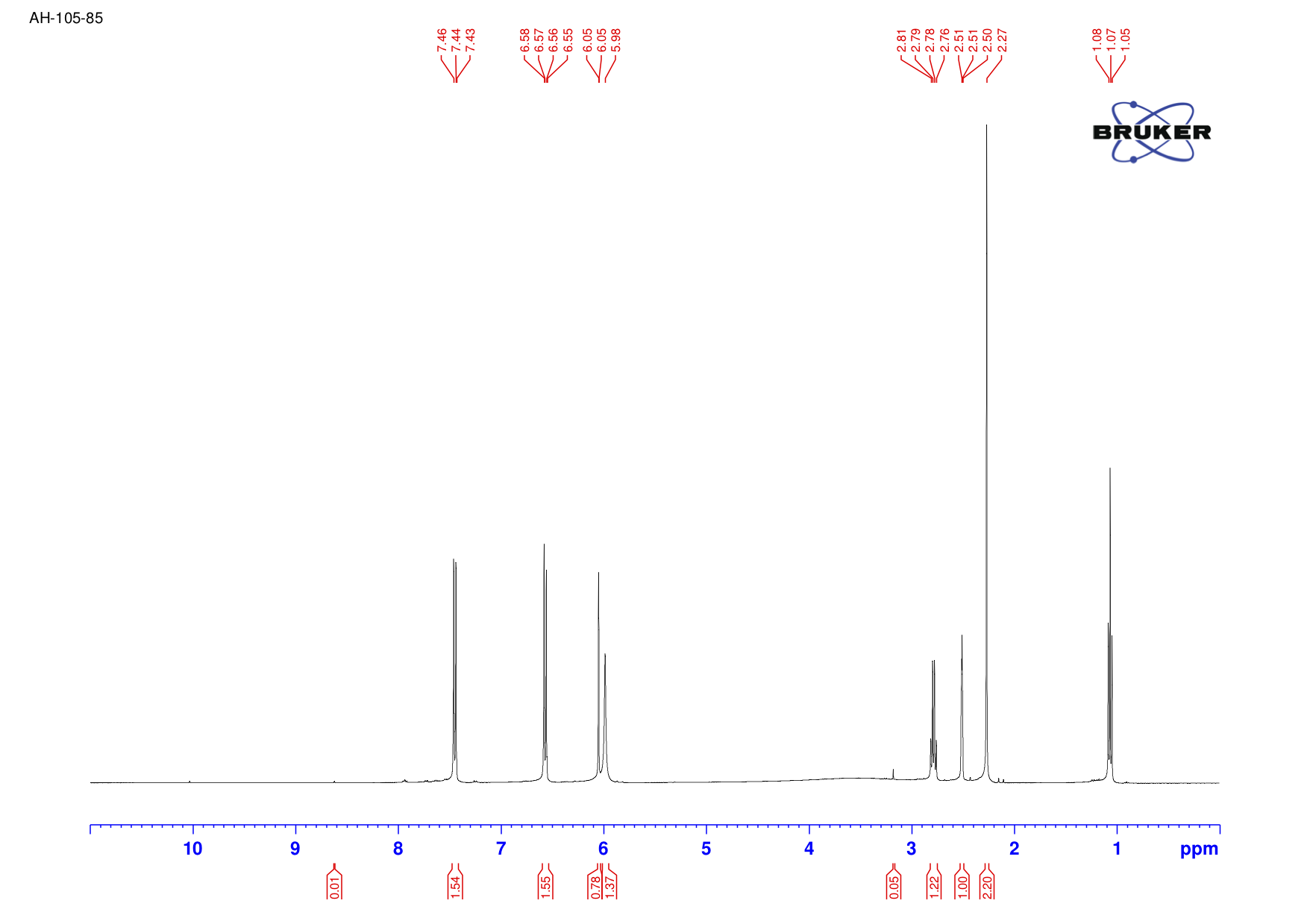


**Figure S1-1**: Proton ^1^H NMR spectrum of Compound **2a.**


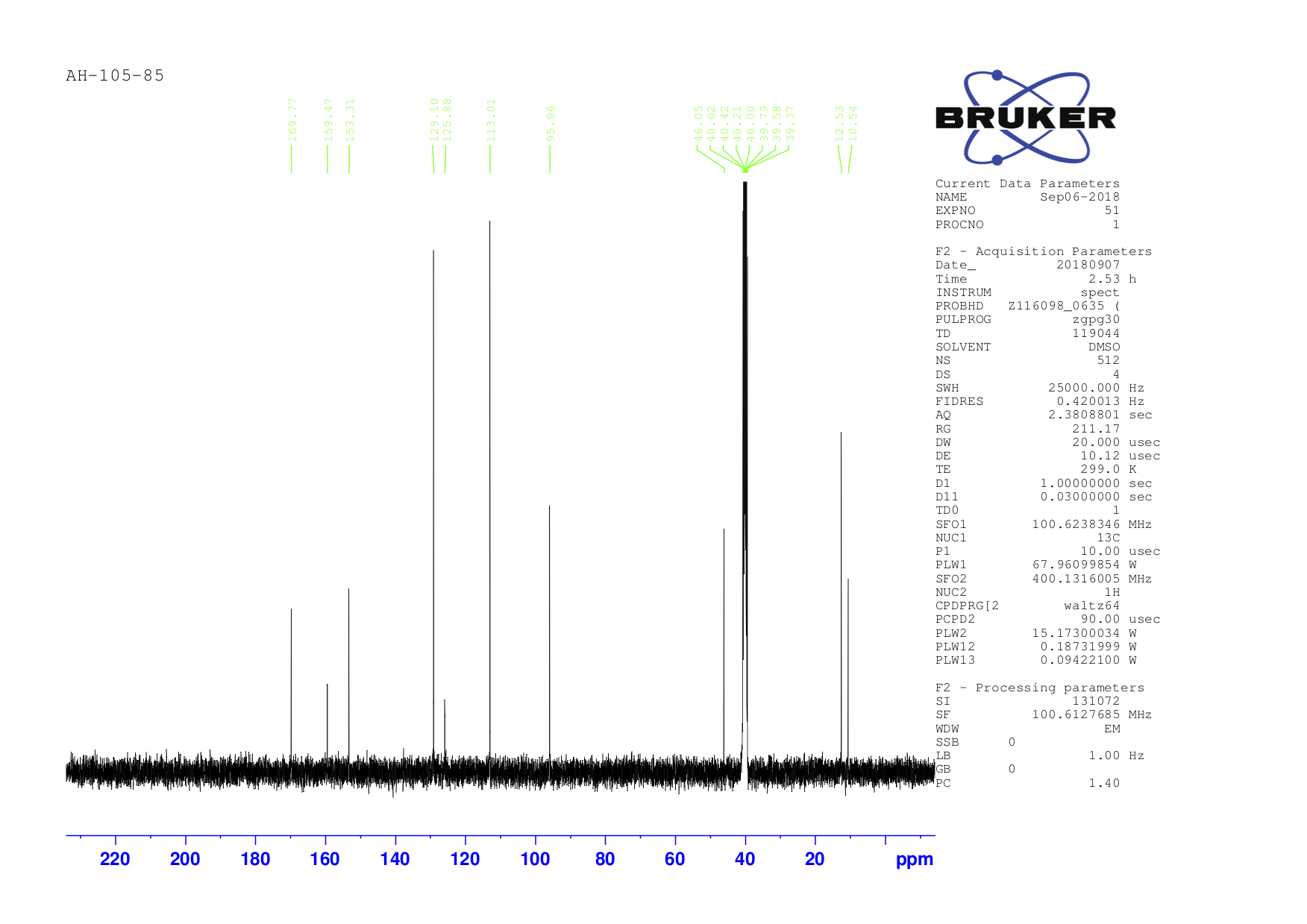


**Figure S1-2**: Carbon ^13^C NMR spectrum of Compound **2a.**

**Figure S1-3**: HRMS spectrum of Compound **2a.**


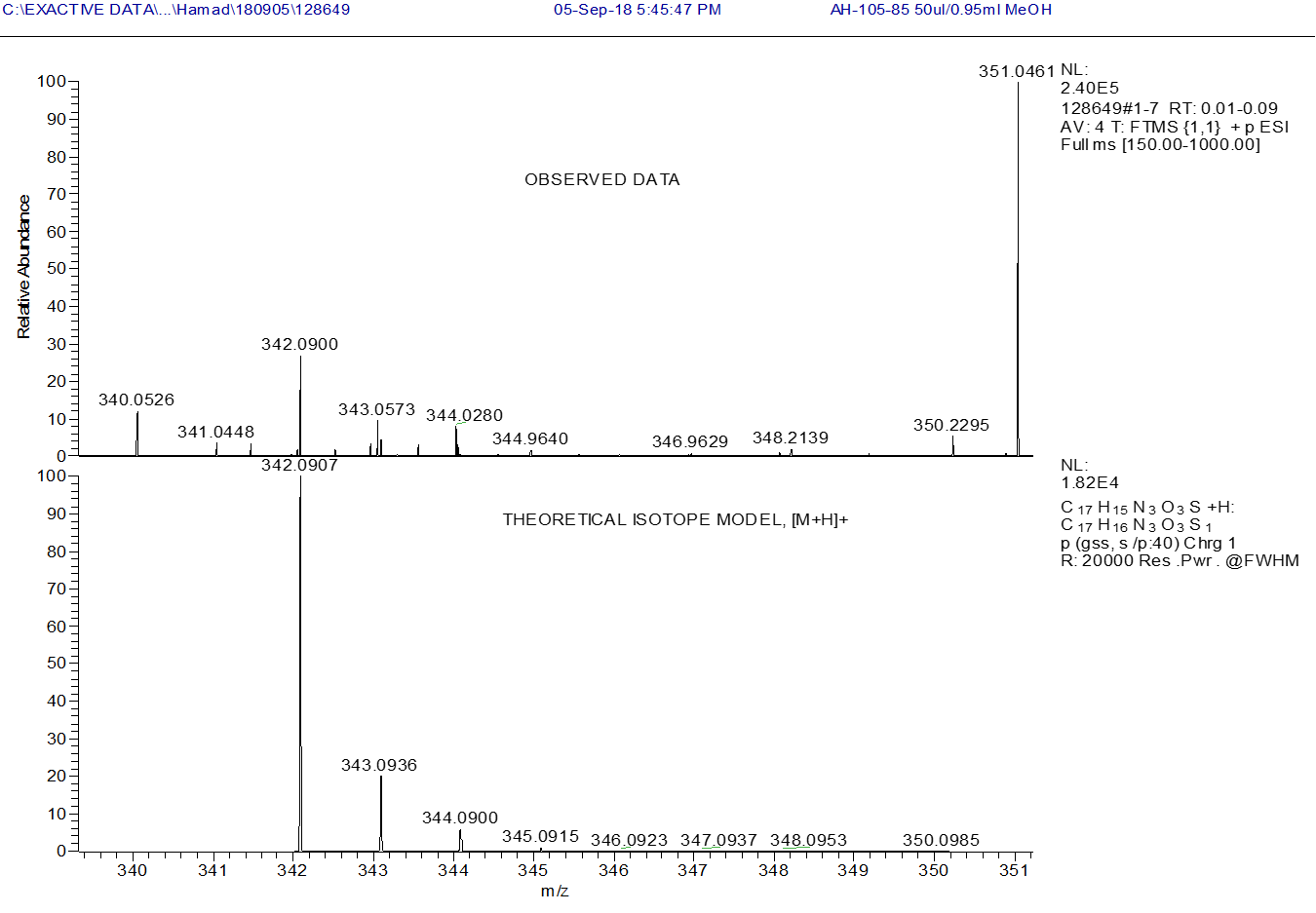


**Figure S1-4**: HRMS spectrum of Compound **2a.**

**4-(4-chloro-2-hydroxybenzylideneamino)-N-(5-methylisoxazol-3-yl)benzenesulfonamide (2b)**

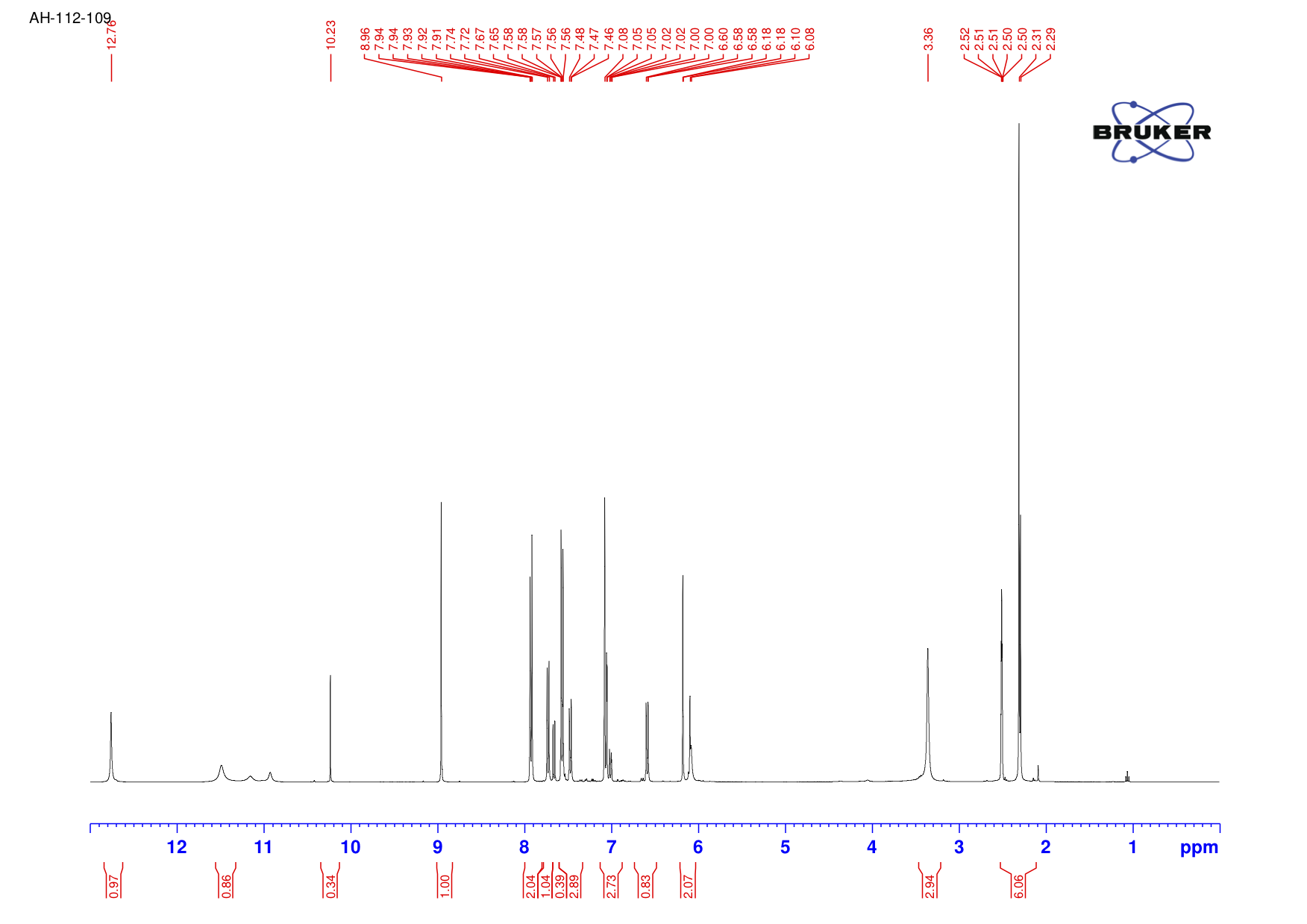


**Figure S2-1**: Proton ^1^H NMR spectrum of Compound **2b.**


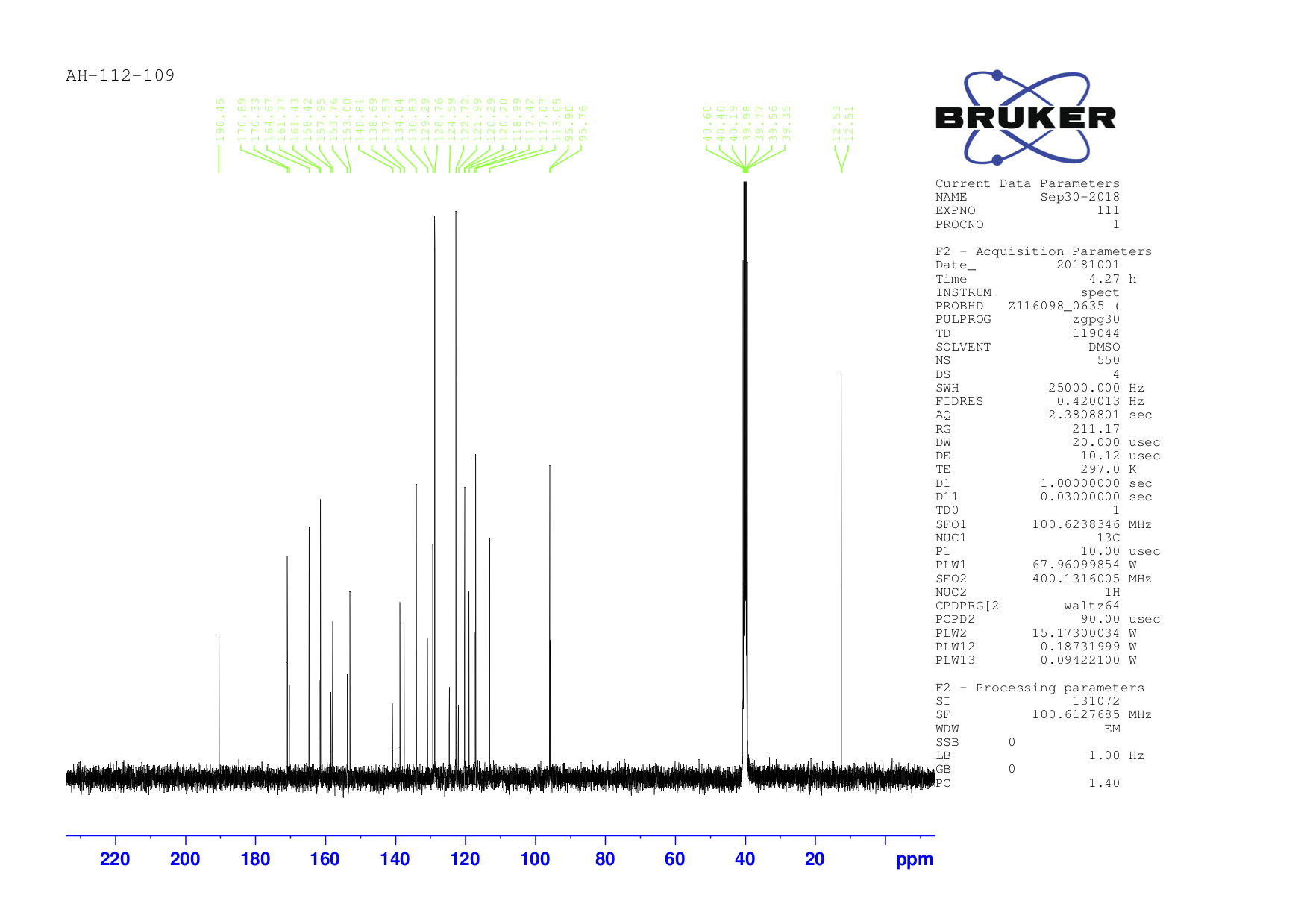


**Figure S2-2**: Carbon ^13^C NMR spectrum of Compound **2b.**

**Figure S2-3**: HRMS spectrum of Compound **2b.**

**Figure S2-4**: HRMS spectrum of Compound **2b.**

**4-{(*E*)-[(4-chlοro-2-hydroxyphenyl)methylidene]amino}-*N*-(6-methοxypyridazin-3-yl)benzene-1-sulfοnamide (2c)**

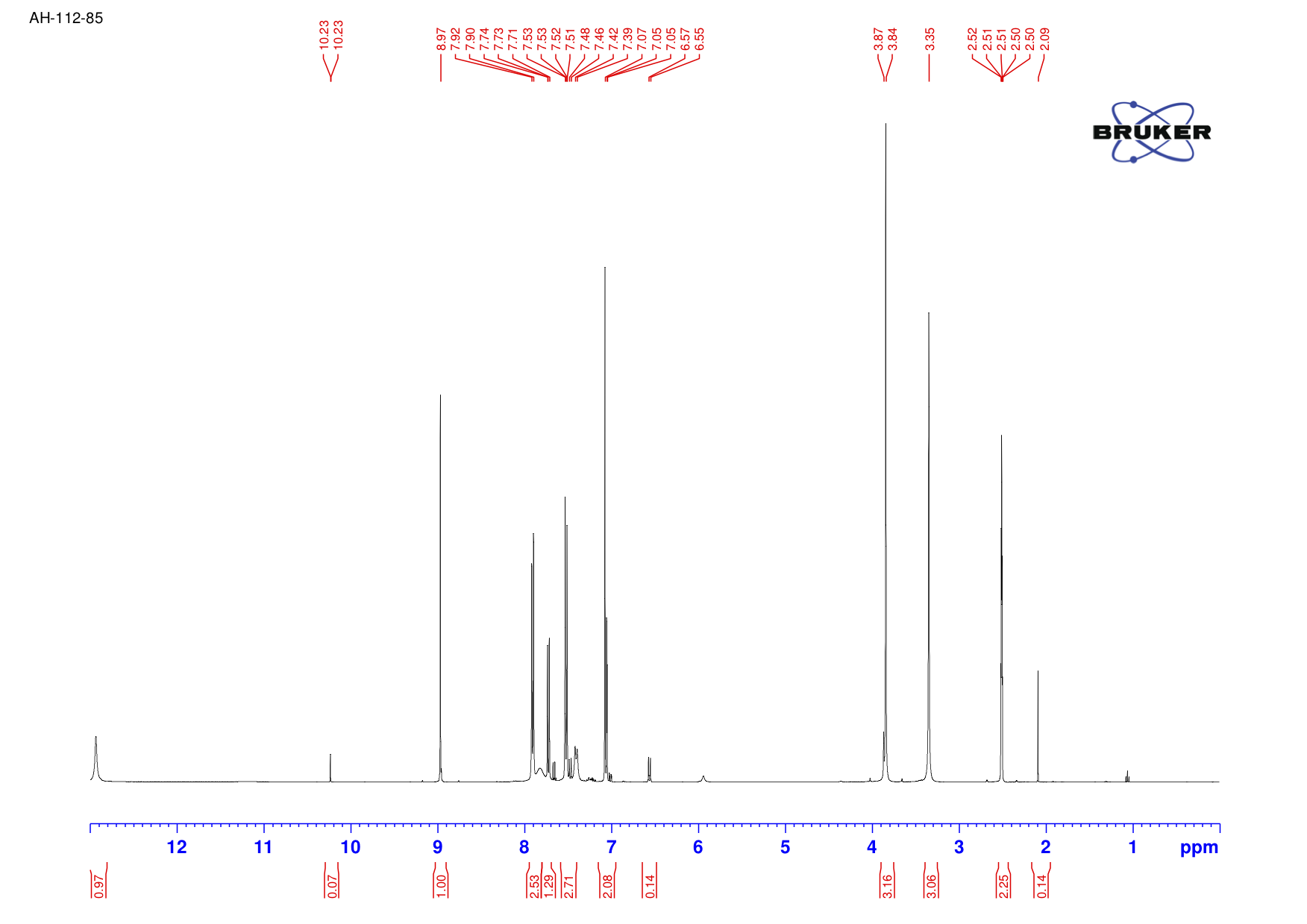


**Figure S3-1**: Proton ^1^H NMR spectrum of Compound **2c.**


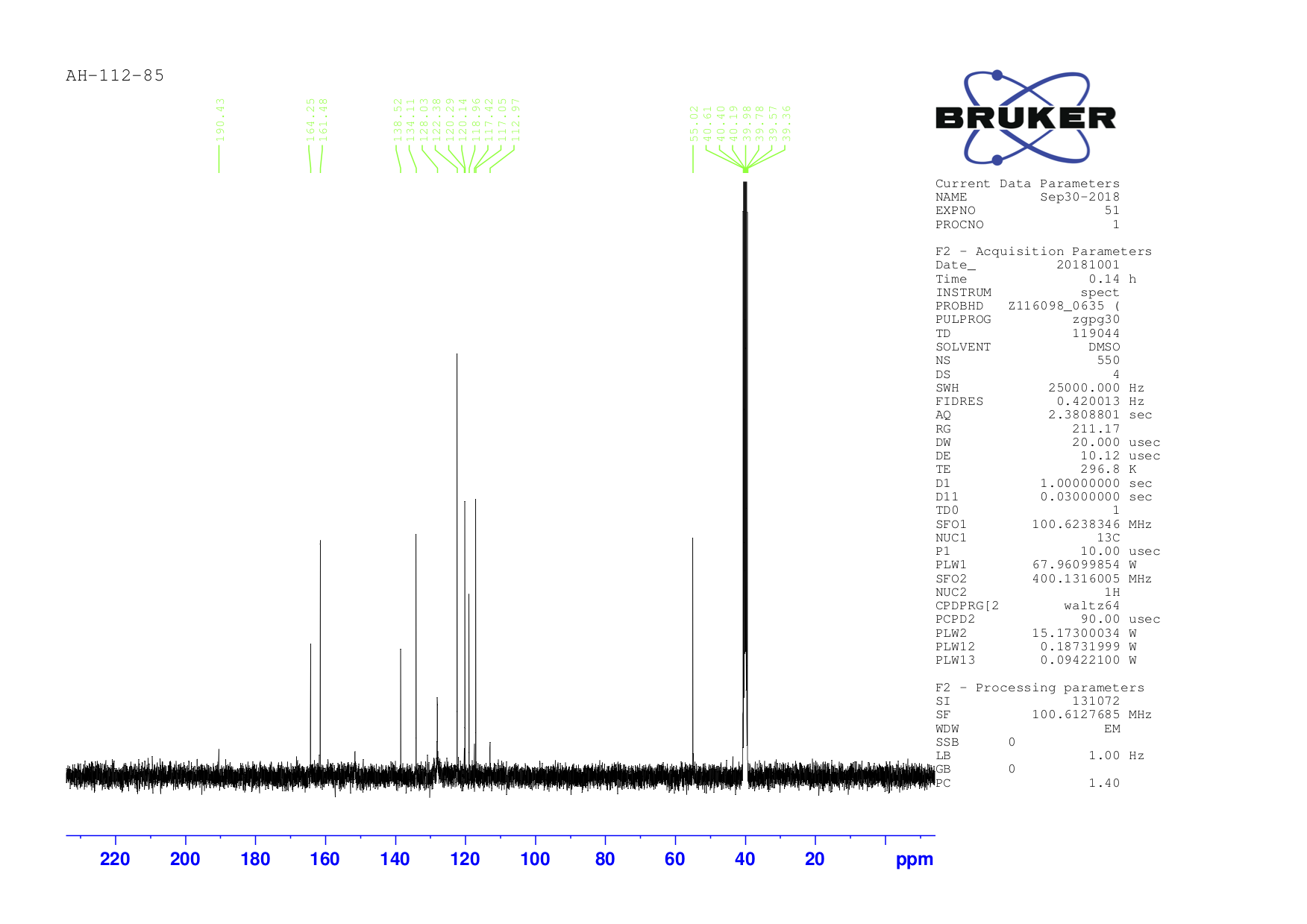


**Figure S3-2**: Carbon ^13^C NMR spectrum of Compound **2c.**

**Figure S3-3**: HRMS spectrum of Compound **2c.**

**Figure S3-4**: HRMS spectrum of Compound **2c.**

**4-{(*E*)-[(4-fluοro-2-hydrοxyphenyl)methylidene]aminο}-*N*-(6-methοxypyridazin-3-yl)benzene-1-sulfοnamide**  **(2d)**

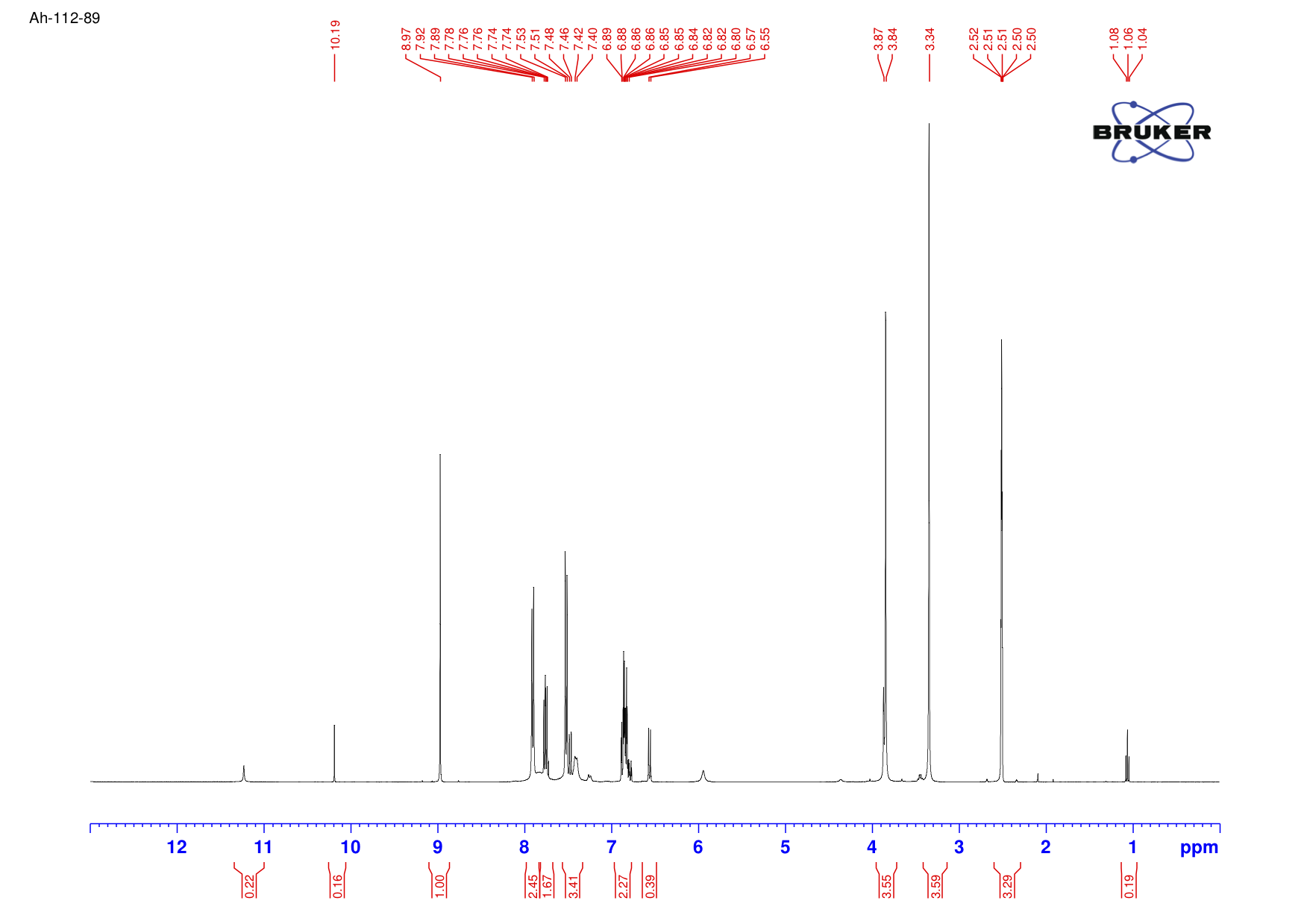


**Figure S4-1**: Proton ^1^H NMR spectrum of Compound **2d.**


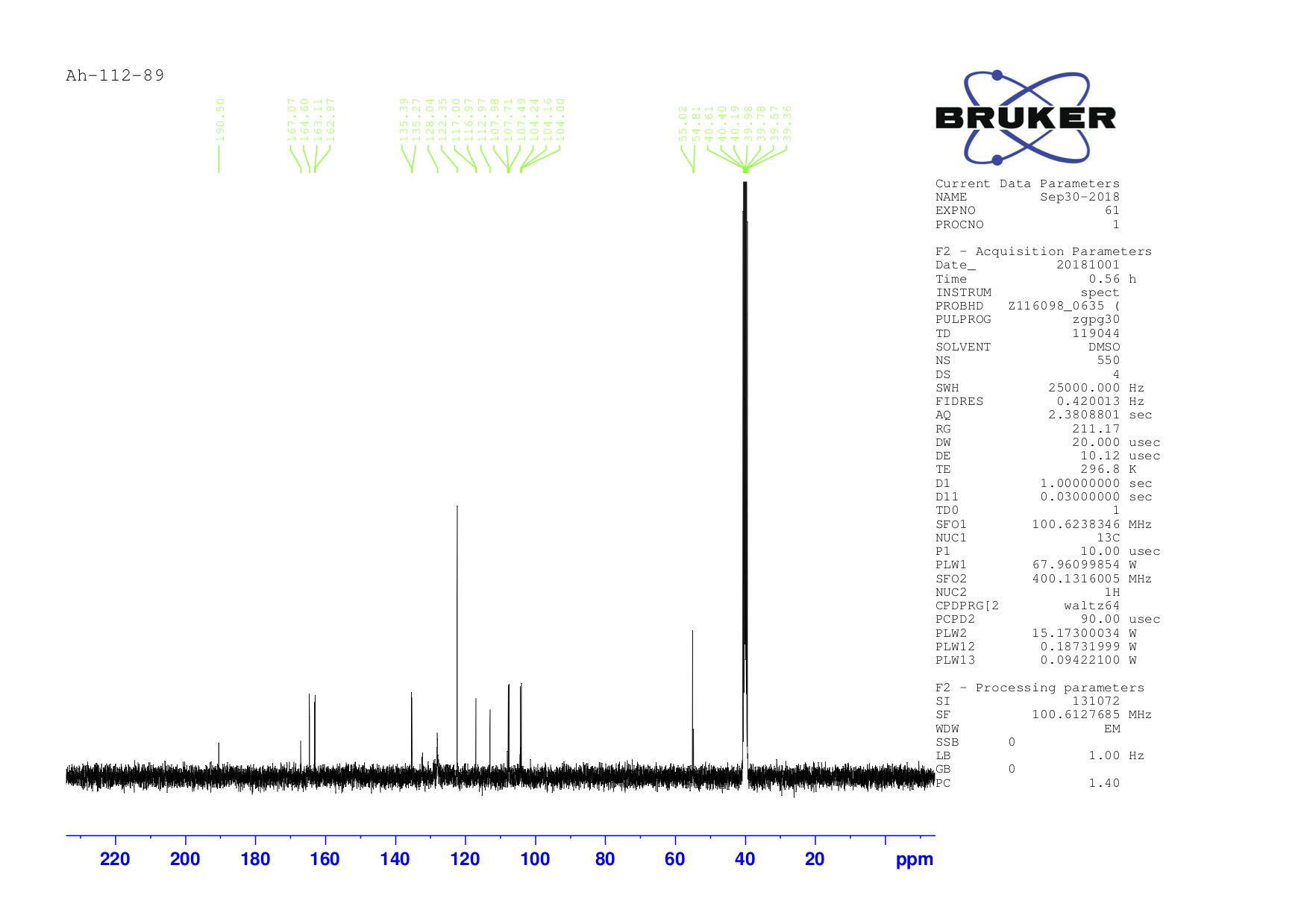


**Figure S4-2**: Carbon ^13^C NMR spectrum of Compound **2d.**

**Figure S4-3**: HRMS spectrum of Compound **2d.**

**Figure S4-4**: HRMS spectrum of Compound **2d.**

**4-{(*E*)-[(2,4-dihydrοxyphenyl)methylidene]aminο}-*N*-(6-methοxypyridazin-3-yl)benzene-1-sulfοnamide (2e)**

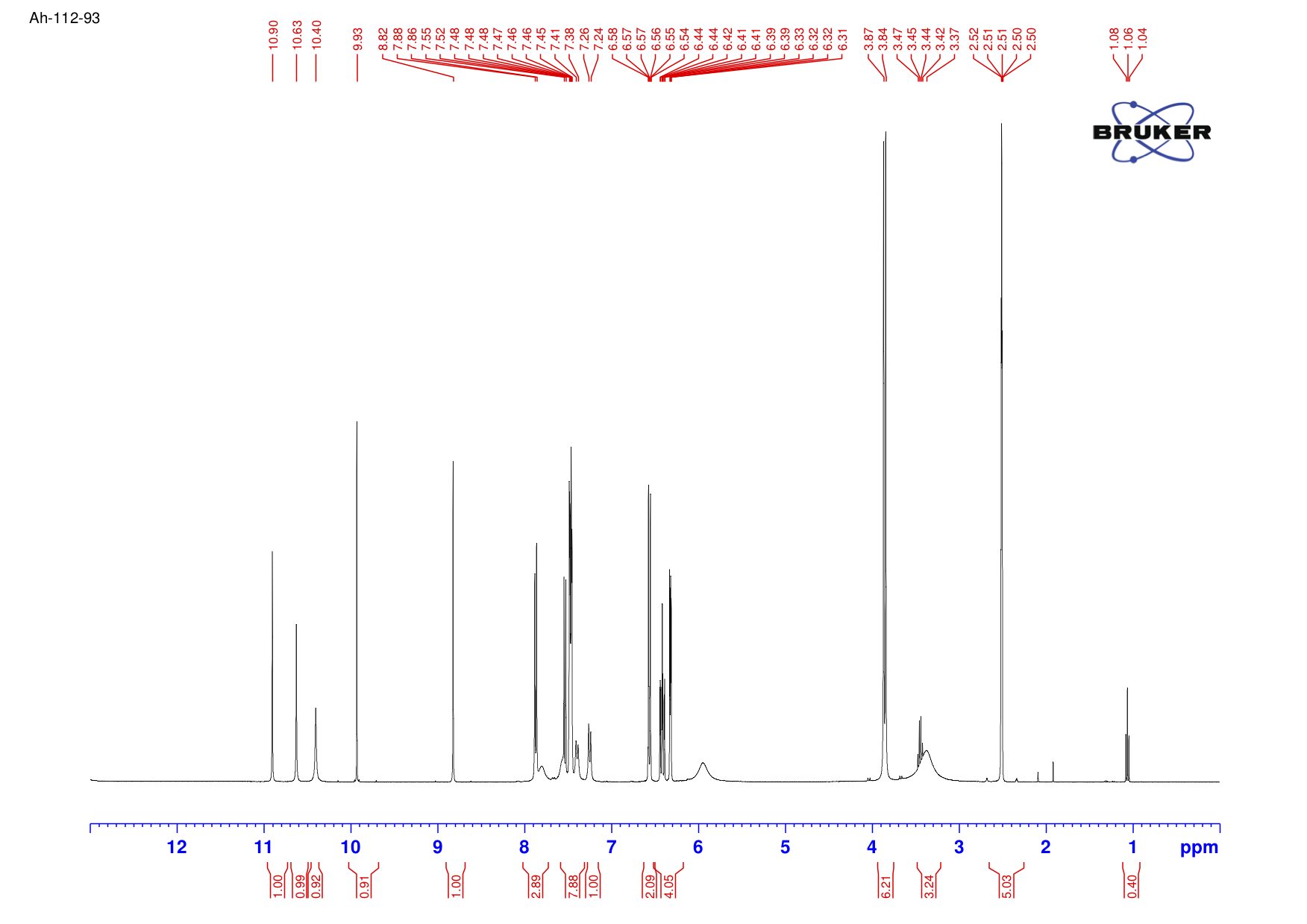


**Figure S5-1**: Proton ^1^H NMR spectrum of Compound **2e.**


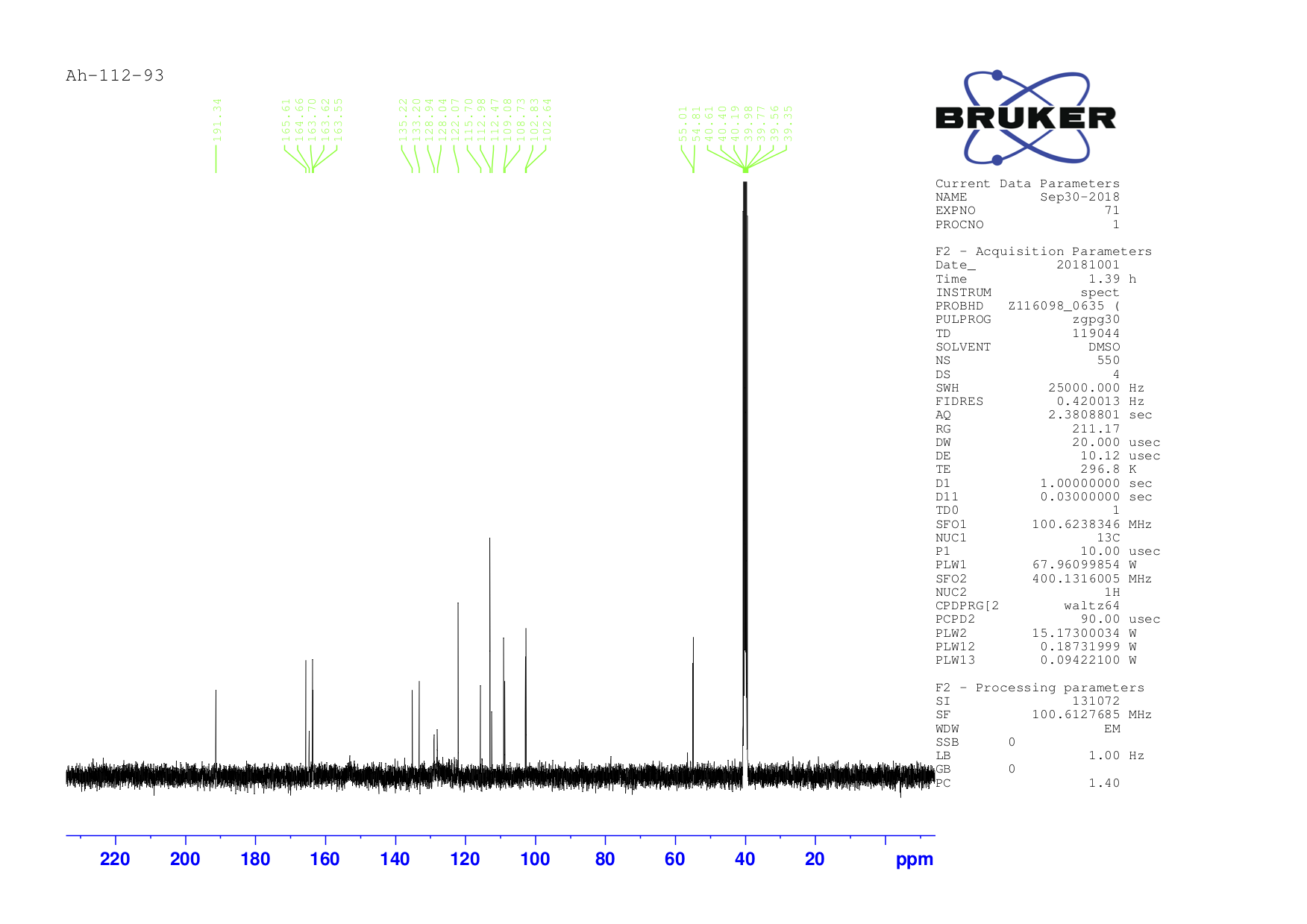


**Figure S5-2**: Carbon ^13^C NMR spectrum of Compound **2e.**

**Figure S5-3**: HRMS spectrum of Compound **2e.**

**Figure S5-4**: HRMS spectrum of Compound **2e.**

**4-{(*E*)-[(2-chloro-4-fluοrophenyl)methylidene]amino}-*N*-(6-methοxypyridazin-3-yl)benzene-1-sulfonamide (2f)**

**
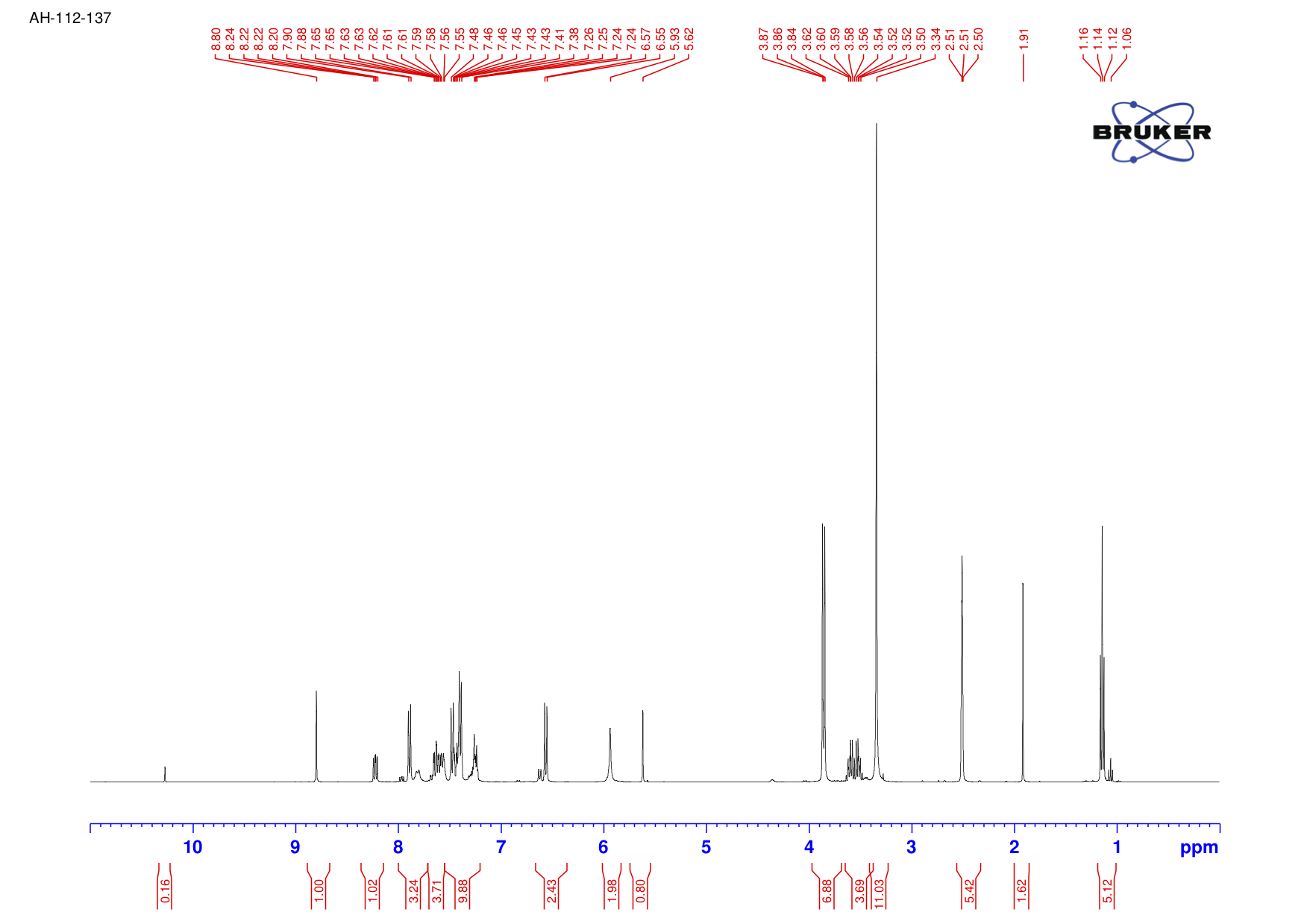
**

**Figure S6-1**: Proton ^1^H NMR spectrum of Compound **2f.**

**
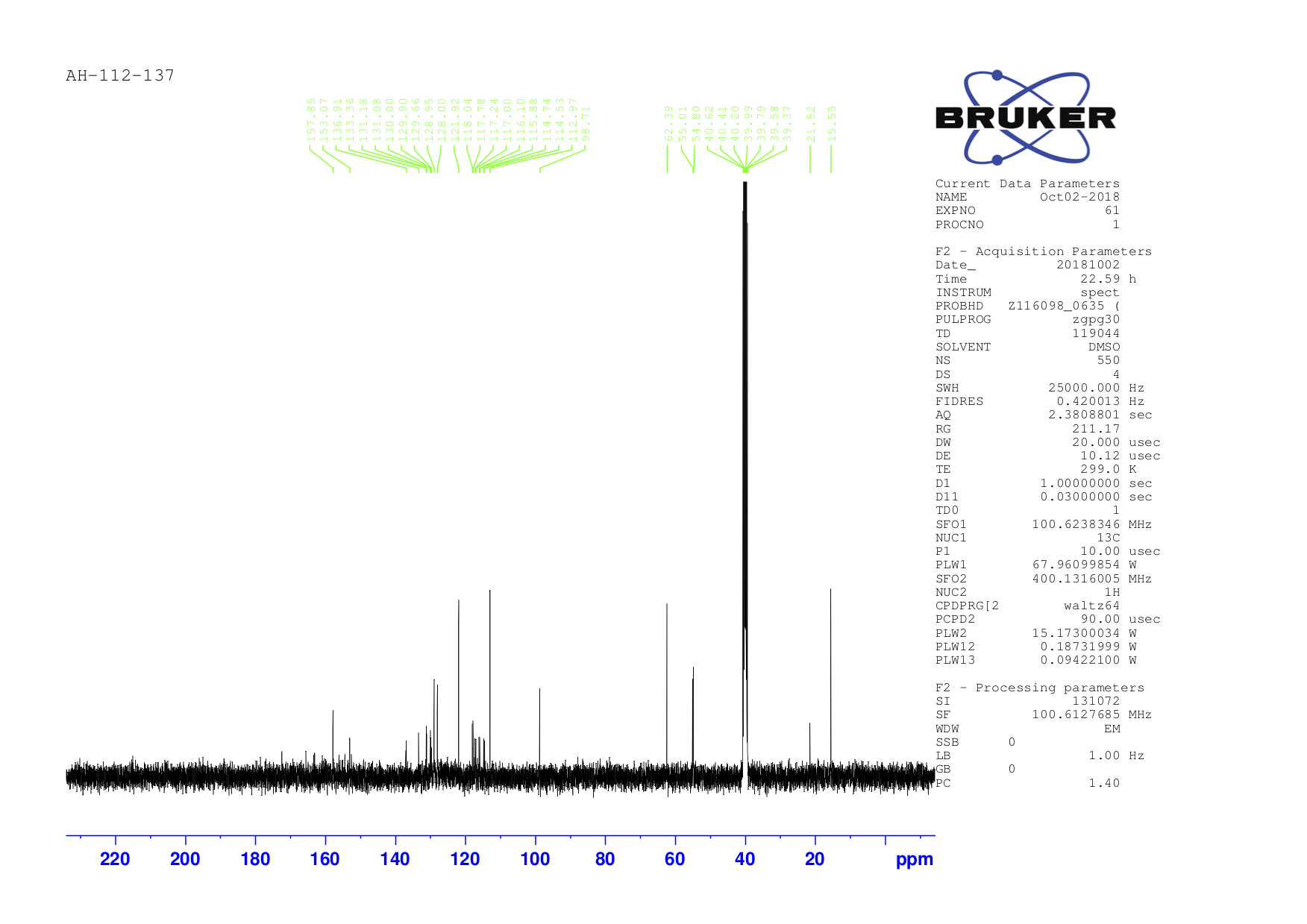
**

**Figure S6-2**: Carbon ^13^C NMR spectrum of Compound **2f.**

**Figure S6-3**: HRMS spectrum of Compound **2f.**

**Figure S6-4**: HRMS spectrum of Compound **2f.**

**4-(5-bromo-2-hydroxybenzylideneamino)-N-(5-methylisoxazol-3-yl)benzenesulfonamide (2g)**

**
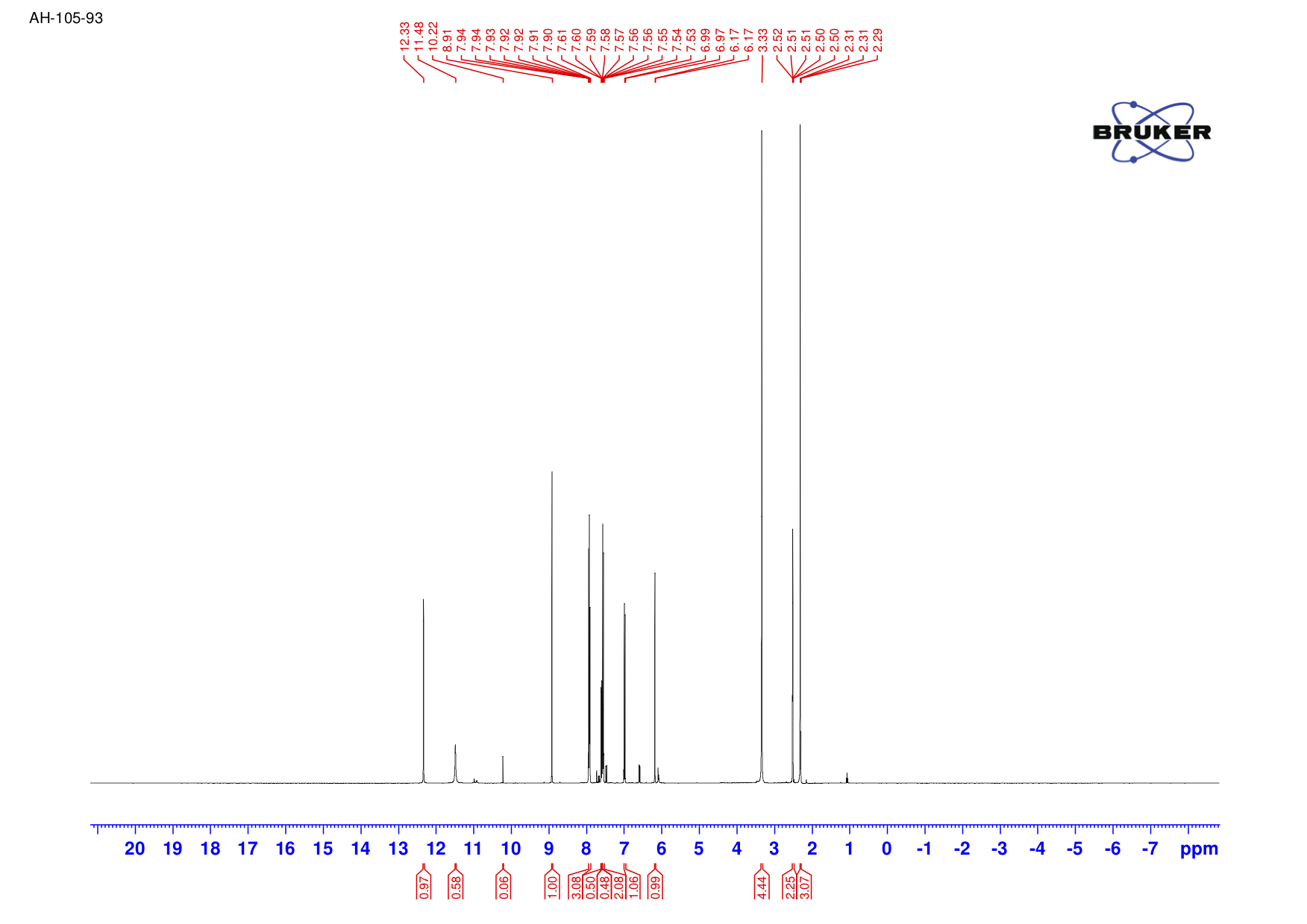
**

**Figure S7-1**: Proton ^1^H NMR spectrum of Compound **2g.**

**
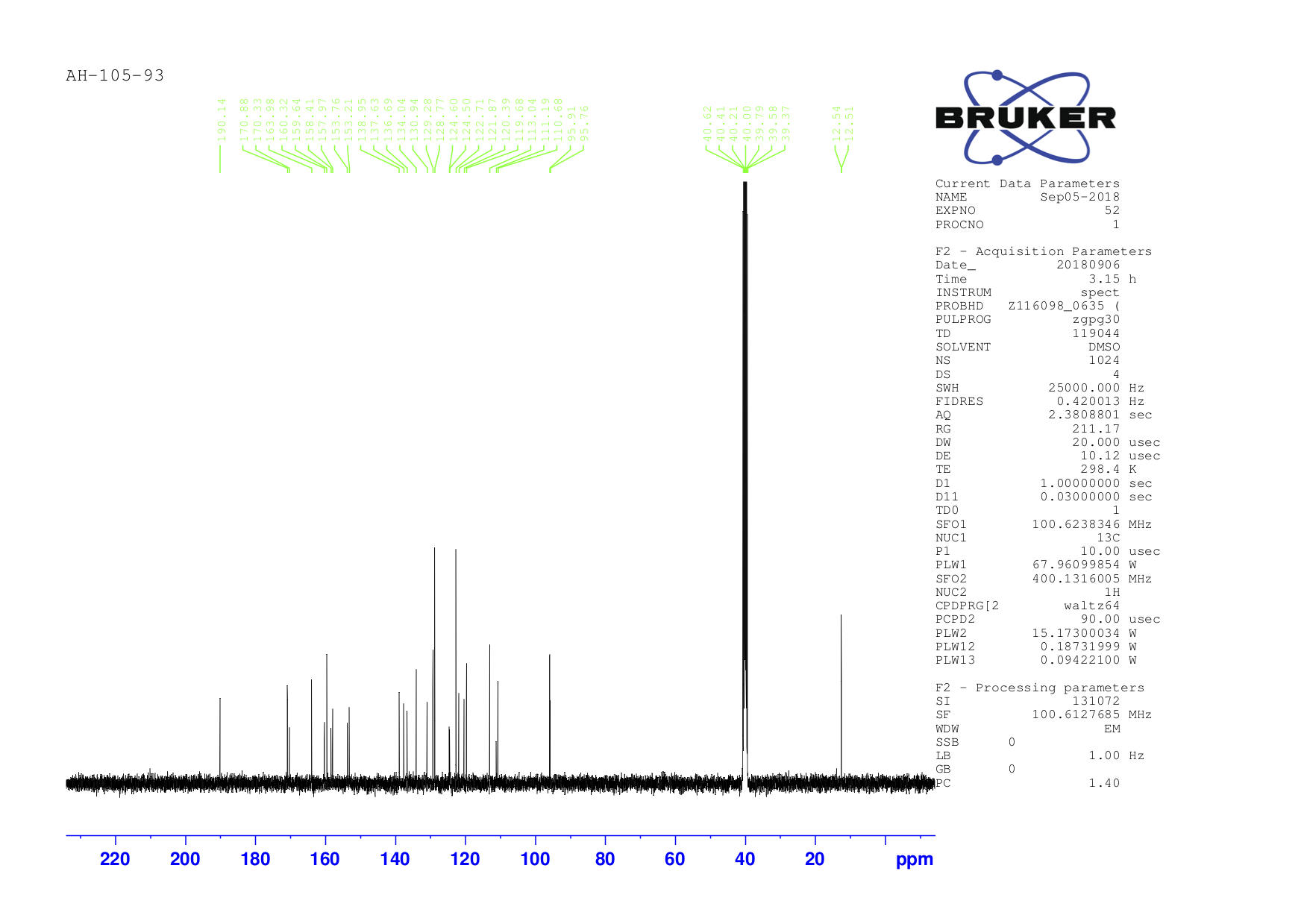
**

**Figure S7-2**: Carbon ^13^C NMR spectrum of Compound **2g.**

**Figure S7-3**: HRMS spectrum of Compound **2g.**

**Figure S7-4**: HRMS spectrum of Compound **2g.**

**4-(5-bromo-2-hydroxybenzylideneamino)-N-(4,6-dimethylpyrimidin-2-yl)benzenesulfonamide (2h)**

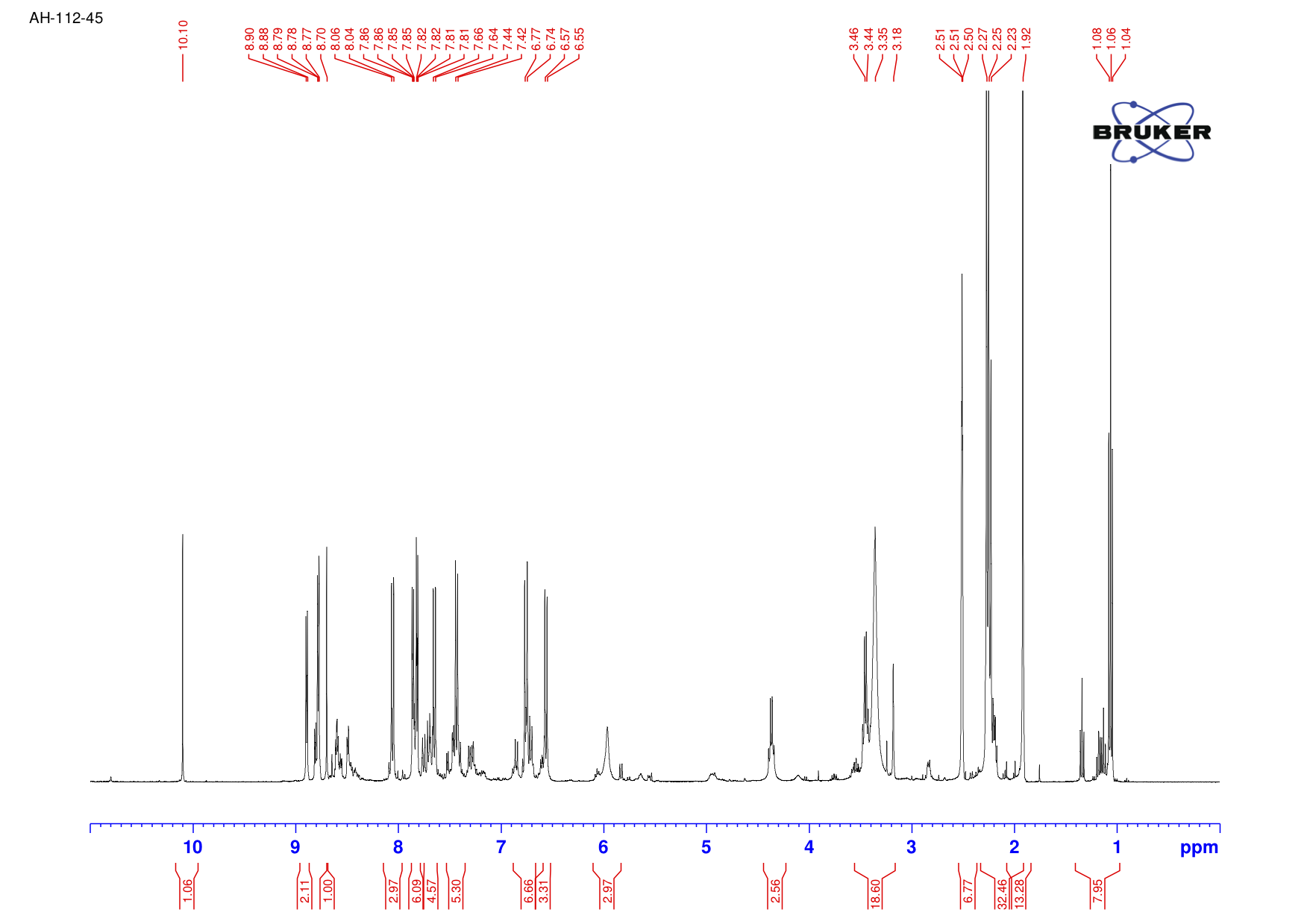


**Figure S8-1**: Proton ^1^H NMR spectrum of Compound **2h.**


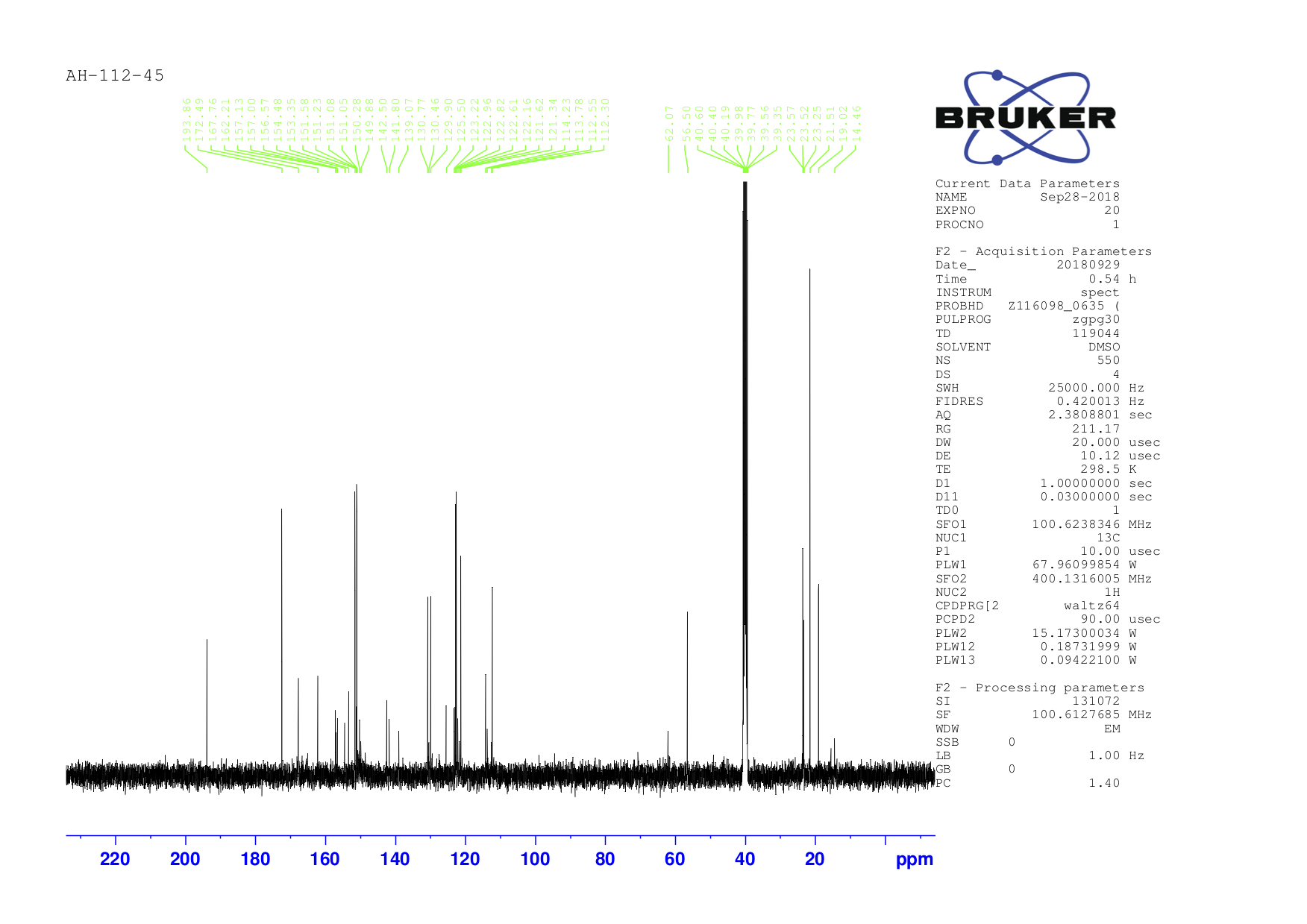


**Figure S8-2**: Carbon ^13^C NMR spectrum of Compound **2h.**

**Figure S8-3**: HRMS spectrum of Compound **2h.**

**Figure S8-4**: HRMS spectrum of Compound **2h.**

**
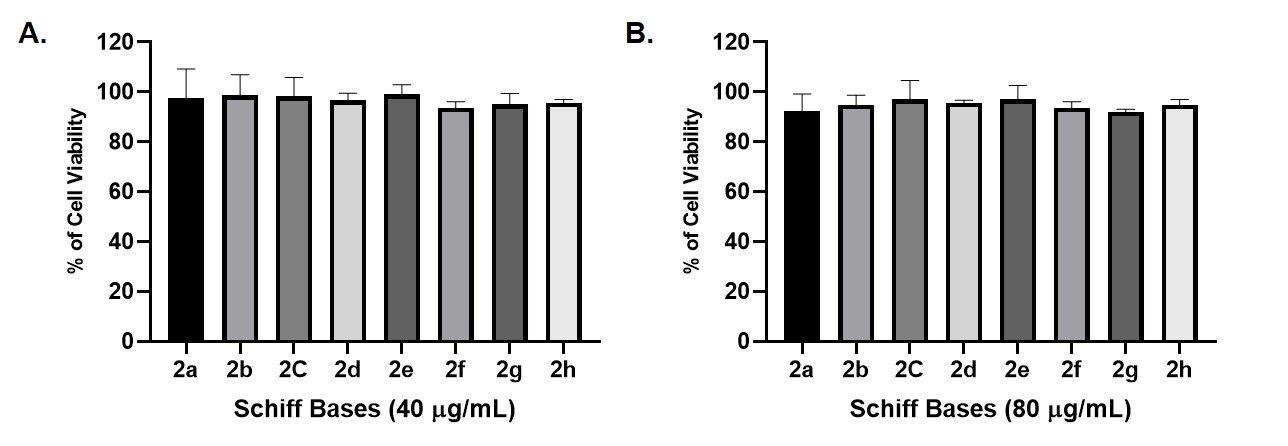
**

**Figure S9: Effect of the Schiff bases 2a to 2h on the viability of the non-tumor cell line WI38 at 40 µg/mL (A) and 80 µg/mL (B) concentrations.**


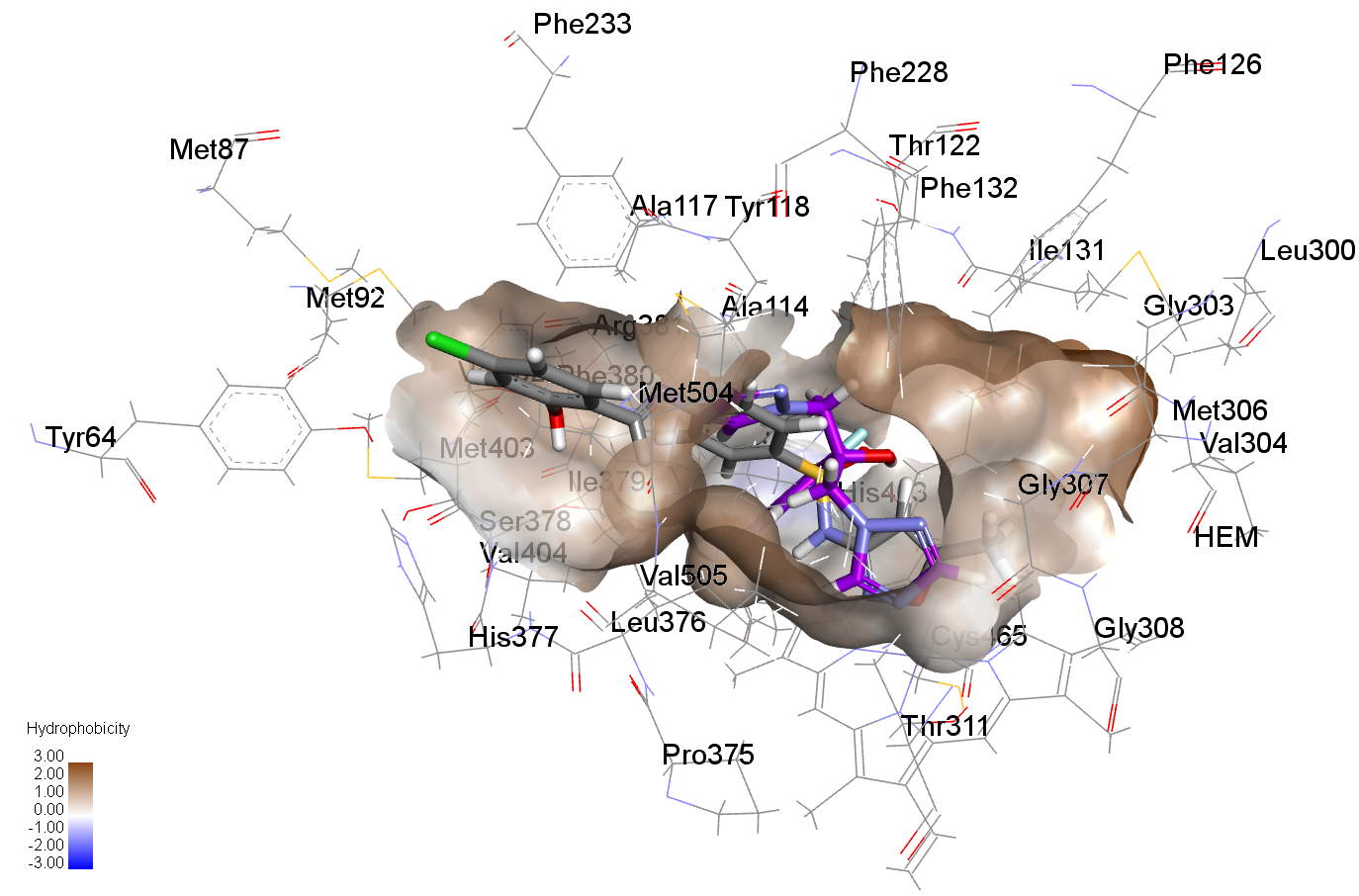


**Figure S10: Fluconazole (cyan) and the Schiff base 2b (grey) at the azole binding pocket of wild type Erg11 from *C. auris***


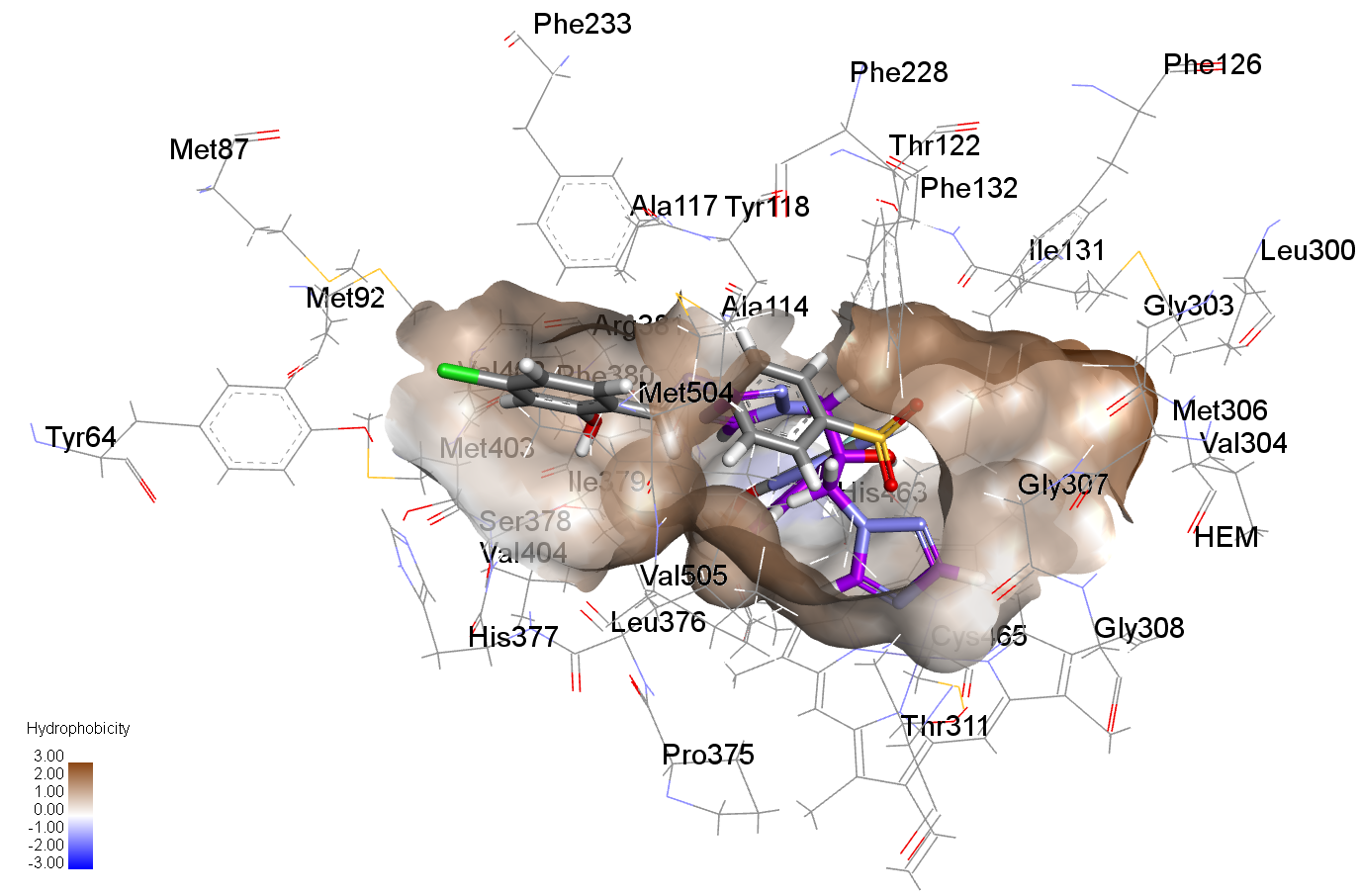


**Figure S11: Fluconazole (cyan) and the Schiff base 2c (grey) at the azole binding pocket of wild type Erg11 from *C. auris***


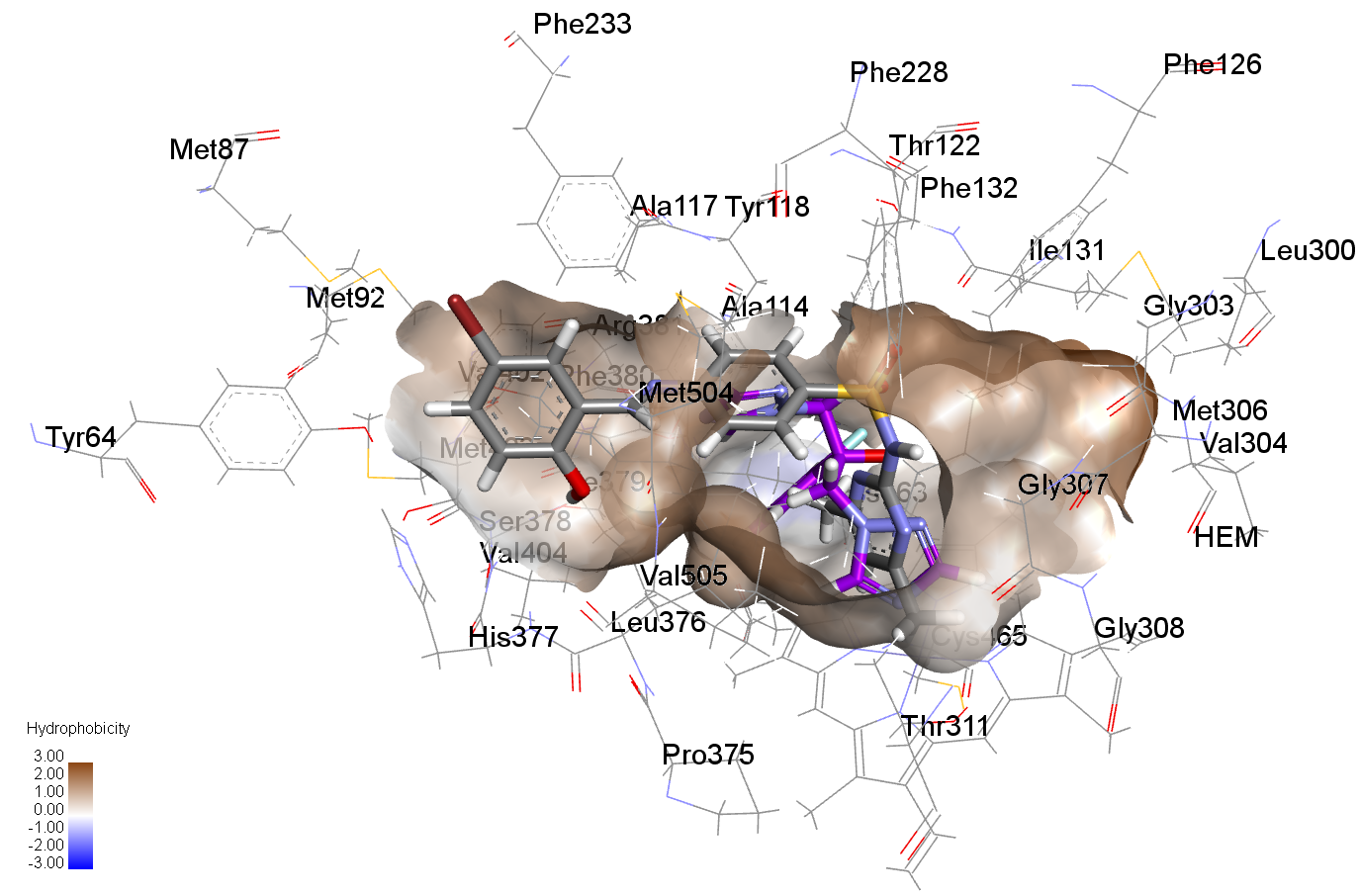


**Figure S11: Fluconazole (cyan) and the Schiff base 2h (grey) at the azole binding pocket of wild type Erg11 from *C. auris***

**
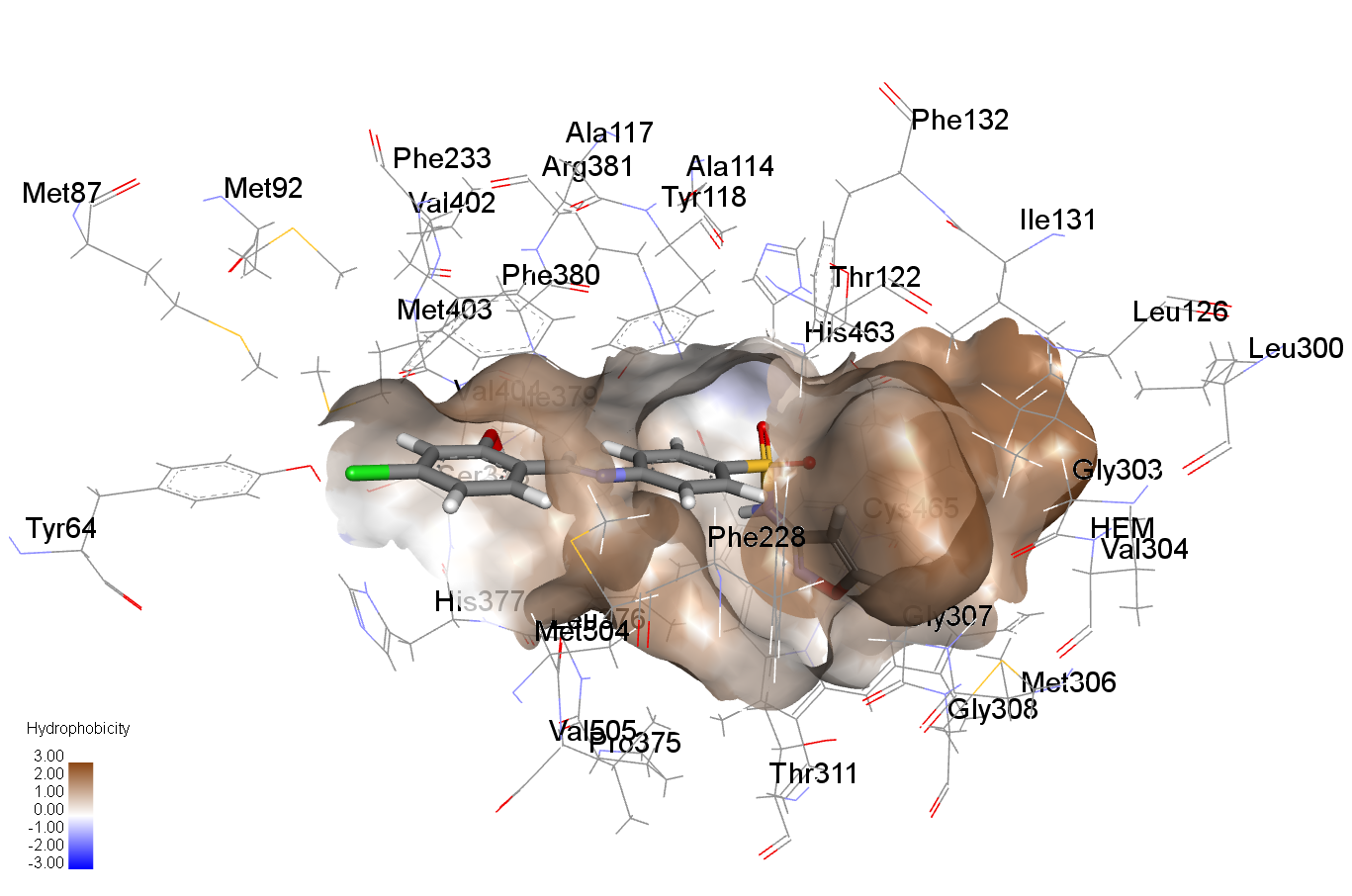
**

**Figure S12: The Schiff base 2b at the azole binding pocket of F126L mutant Erg11 from *C. auris***

**
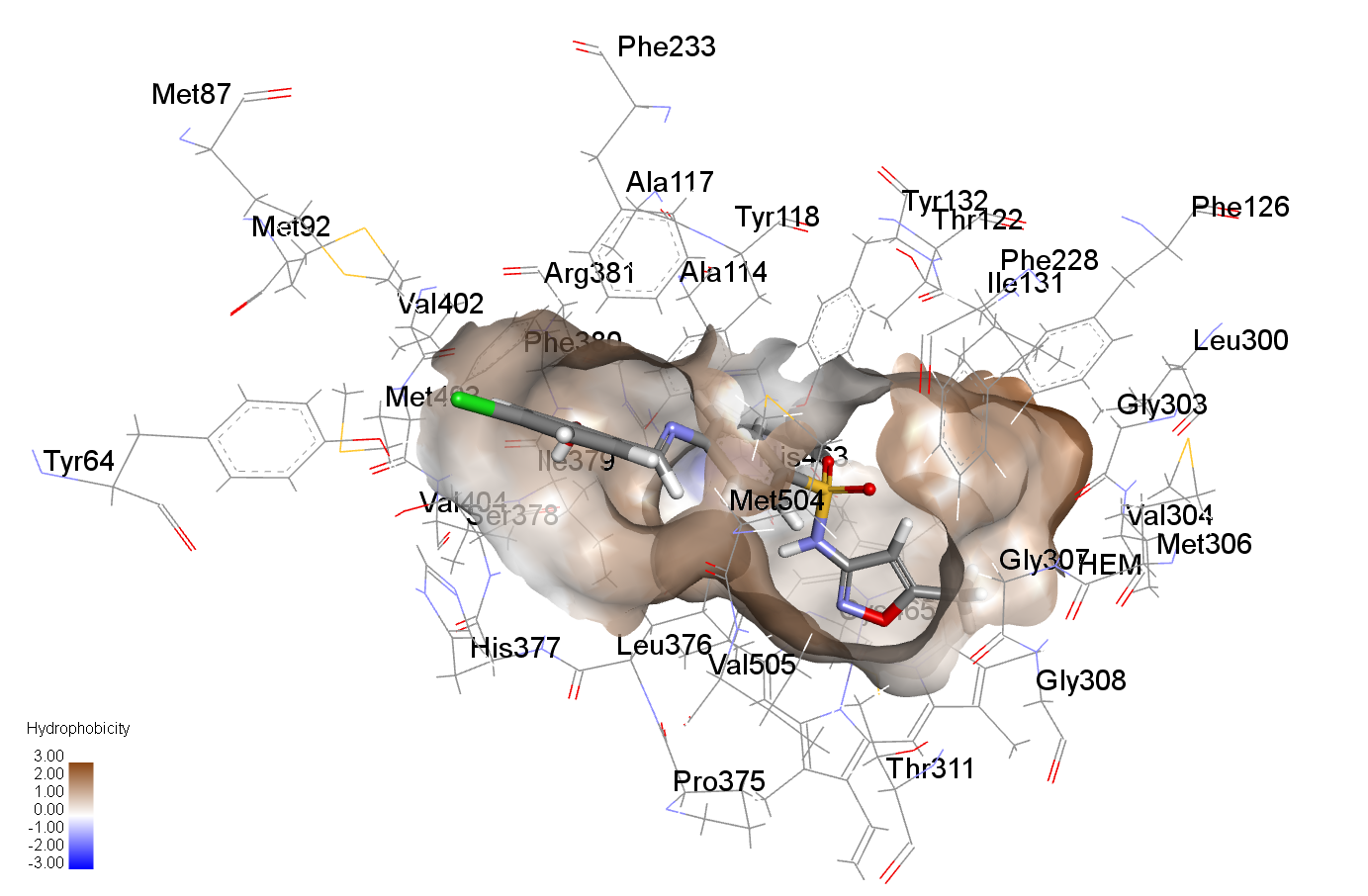
**

**Figure S12: The Schiff base 2b at the azole binding pocket of F132Y mutant Erg11 from *C. auris***
